# Allergen-specific mRNA–lipid nanoparticle therapy for prevention and treatment of experimental allergy in mice

**DOI:** 10.1172/JCI194080

**Published:** 2025-09-23

**Authors:** Yrina Rochman, Michael Kotliar, Andrea M. Klingler, Mark Rochman, Mohamad-Gabriel Alameh, Jilian R. Melamed, Garrett A. Osswald, Julie M. Caldwell, Jennifer M. Felton, Lydia E. Mack, Julie Hargis, Ian P. Lewkowich, Artem Barski, Drew Weissman, Marc E. Rothenberg

**Affiliations:** 1 Division of Allergy and Immunology, Cincinnati Children’s Hospital Medical Center, Cincinnati, Ohio, USA.; 2 Department of Medicine, University of Pennsylvania, Philadelphia, Pennsylvania, USA.; 3 Department of Pathology and Laboratory Medicine, Children’s Hospital of Philadelphia, Pennsylvania, USA.; 4 Division of Immunobiology, Cincinnati Children’s Hospital Medical Center, Cincinnati, Ohio, USA.; 5 Department of Pediatrics, College of Medicine, University of Cincinnati, Cincinnati, Ohio, USA.; 6 Division of Human Genetics, Cincinnati Children’s Hospital Medical Center, Cincinnati, Ohio, USA.

**Keywords:** Immunology, Inflammation, Adaptive immunity, Allergy, Immunotherapy

## Abstract

Allergic diseases have reached epidemic proportions globally, calling attention to the need for better treatment and preventive approaches. Herein, we developed allergen-encoding messenger RNA (mRNA)–lipid nanoparticle (LNP) strategies for both therapy and prevention of allergic responses. Immunization with allergen-encoded mRNA-LNPs modulated T cell differentiation, inhibiting the generation of T helper type 2 and type 17 cells upon allergen exposure in experimental asthma models induced by ovalbumin, and naturally occurring house dust mite (HDM) and the major HDM allergen Der p1. Allergen-specific mRNA-LNP treatment attenuated clinicopathology in both preventive and established allergy models, including reduction in eosinophilia, mucus production, and airway hypersensitivity, while enhancing production of allergen-specific IgG antibodies and maintaining low IgE levels. Additionally, allergen-specific mRNA-LNP vaccines in mice elicited a CD8^+^CD38^+^KLRG^–^ T cell response as seen following SARS-CoV-2 mRNA vaccination in humans, underscoring a conserved immune mechanism across species, regardless of the mRNA-encoded protein. Notably, mRNA-LNP vaccination in combination with an mTOR inhibitor reduced the CD8^+^ T cell response without affecting the vaccine-induced anti-allergic effect in the preventive model of asthma. This technology renders allergen-specific mRNA-LNP therapy a promising approach for prevention and treatment of allergic diseases.

## Abstract 2

Allergen-specific mRNA-LNP therapy modulates immune responses, reducing allergy symptoms while enhancing protective immunity, offering a promising strategy for both prevention and treatment of allergic diseases.

## Graphical abstract

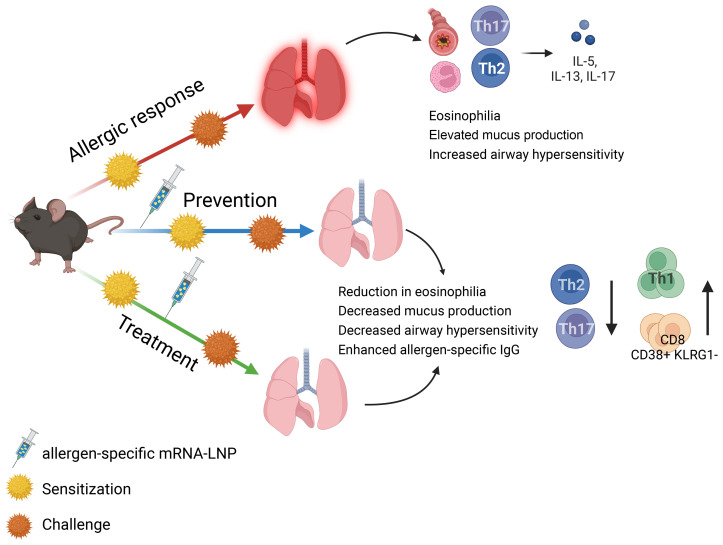

## Introduction

Allergic diseases have reached epidemic proportions, affecting nearly 30% of the worldwide population (1–3). Despite substantial advances in treating these diseases, unchecked allergic inflammation remains a leading cause of hospital admission, morbidity, and even mortality in children and adults. Current therapeutic approaches focus on anti-cytokines, anti-cytokine receptors, anti-IgE, and antiinflammatory mediators like glucocorticoids (4–6). Another approach to treat allergies focuses on immunotherapy, which is currently limited to prolonged courses of subcutaneous or sublingual administration of allergen extracts; yet this approach is typically only effective for nasal or ocular allergies (e.g., allergic rhinoconjunctivitis) (7, 8). Recently, oral immunotherapy for food allergy, consisting of slowly reintroducing foods, has been shown to be effective in some individuals (9–11). Recombinant allergens, hypoallergenic variants, epitope-based immunotherapies, allergen-encoding plasmid DNA and messenger RNA (mRNA), and adjuvant-based immunomodifiers, such as non-methylated CG-rich DNA sequences, bacteria, or their extracts, are under development and hold promise (7, 8, 12–19).

The application of nucleoside-modified mRNA vaccines encapsulated within lipid nanoparticles (LNPs) has exhibited remarkable efficacy in curtailing viral and bacterial infections (20–24) and is promising for other diseases ranging from cancer to autoimmunity (25–28). Nucleoside-modified mRNA vaccines possess several advantages over conventional counterparts: they induce rapid and highly specific immune responses, are versatile in terms of the encoded immunogen, can be manufactured with high quality under controlled conditions, are generally safe owing to the absence of viral components and integration into host DNA, and are cost-effective (29–31). Importantly, encapsulating the nucleoside-modified mRNA within LNPs removes the necessity for additional adjuvants to provoke immune responses, given that LNPs intrinsically exhibit adjuvant properties (32–34). LNPs provide protection against mRNA degradation, enabling administration of lower mRNA doses while maintaining robust efficacy (21, 34–36).

Engineered against infectious diseases and cancer, mRNA-LNP vaccines stimulate an immune response by fostering the generation of T helper type 1 (Th1), T follicular helper (Tfh), and cytotoxic CD8^+^ T cells, accompanied by IgG antibodies (21, 28, 35, 37, 38). It has been demonstrated that the antigen-specific mRNA-LPX (liposome) vaccine stimulates regulatory T cells (Tregs), offering protection against experimental autoimmune encephalomyelitis (27). In addition, targeting mRNA-LNPs to tolerogenic liver sinusoidal endothelial cells by decorating the LNP with mannose ligands has been shown to be effective against peanut-induced anaphylaxis (39). These collective findings form the foundation for our hypothesis that nucleoside-modified allergen-specific mRNA-LNP vaccines, by shaping CD4^+^ and CD8^+^ T cell responses and inducing allergen-specific IgG1 and IgG2 antibodies, could block Th2 cell activation and create an anti-allergic environment, thereby preventing manifestations of allergy including experimental asthma.

## Results

### Allergen-specific mRNA-LNP immunization reduces allergic responses in experimental asthma.

We hypothesized that the ability of allergen-specific mRNA-LNPs to induce a Th1 response and increase the level of antigen-specific IgGs (21, 22, 38) could be utilized for protection against allergic responses. To test this hypothesis, we employed an allergy model using the egg antigen ovalbumin (OVA), as tools existed for detailed examination of allergen-specific immune responses. First, we tested the efficiency and specificity of the nucleoside-modified OVA-mRNA-LNPs in inducing T cell responses in mice engineered to express T cells with OVA-specific receptors (OTII cells). Mice were injected with purified naive OTII cells expressing the CD45.1 marker and treated intramuscularly (i.m.) with *N*1-methylpseudouridine (m1Ψ)–mRNA–LNPs encoding OVA protein (OVA-mRNA-LNP), empty LNP, or phosphate-buffered saline (PBS) twice on days 0 and 7 (Supplemental Figure 1A; supplemental material available online with this article; https://doi.org/10.1172/JCI194080DS1). The response of antigen-specific CD4^+^ T cells in lymph nodes (LNs) was analyzed 4 days later. The administration of OVA-mRNA-LNP induced marked expansion of donor OTII cells, increased the activation marker CD44, and enhanced the production of TNF-α and IL-2 cytokines in the local LNs, indicating an efficient and antigen-specific T cell response to the vaccine (Supplemental Figure 1B). There were an increase in the proportion of donor OTII cells and host CD4^+^ T cells producing IFN-γ and expressing T-bet, which are key characteristics of Th1 cells, and an elevation in percentages of Bcl6^+^PD-1^+^ cells, indicative of Tfh cells (Supplemental Figure 1C), that were in line with previous reports (32, 37, 40). We did not observe elevation in IL-17A, IL-4, or Foxp3 levels (Supplemental Figure 1D) in response to OVA-mRNA-LNP. No activation of host or OTII T cells was detected following treatment with PBS or empty LNP.

To assess the dose range of allergen-specific mRNA-LNP required for effective T cell activation, mice receiving donor OTII cells were treated with a single i.m. injection of increasing amounts of OVA-mRNA-LNP. By day 7, the analysis of local LNs showed strong OTII cell expansion at doses of 2–5 μg of OVA-mRNA-LNP, with over 80% of the cells expressing the activation marker CD44 (Supplemental Figure 2A). These doses also correlated with increased frequencies of effector OTII cells producing TNF-α or IFN-γ or exhibiting a Bcl6^+^PD-1^+^ phenotype (Supplemental Figure 2B). Concurrently with the T cell response, a dose-dependent increase in OVA-specific IgG1, IgG2a, and IgG2b levels was observed in serum 18 days after treatment (Supplemental Figure 2C). These results demonstrate the specificity and high potency of the OVA-mRNA-LNP in activating and differentiating Th cells toward Th1 and Tfh subsets.

**Figure 1:**
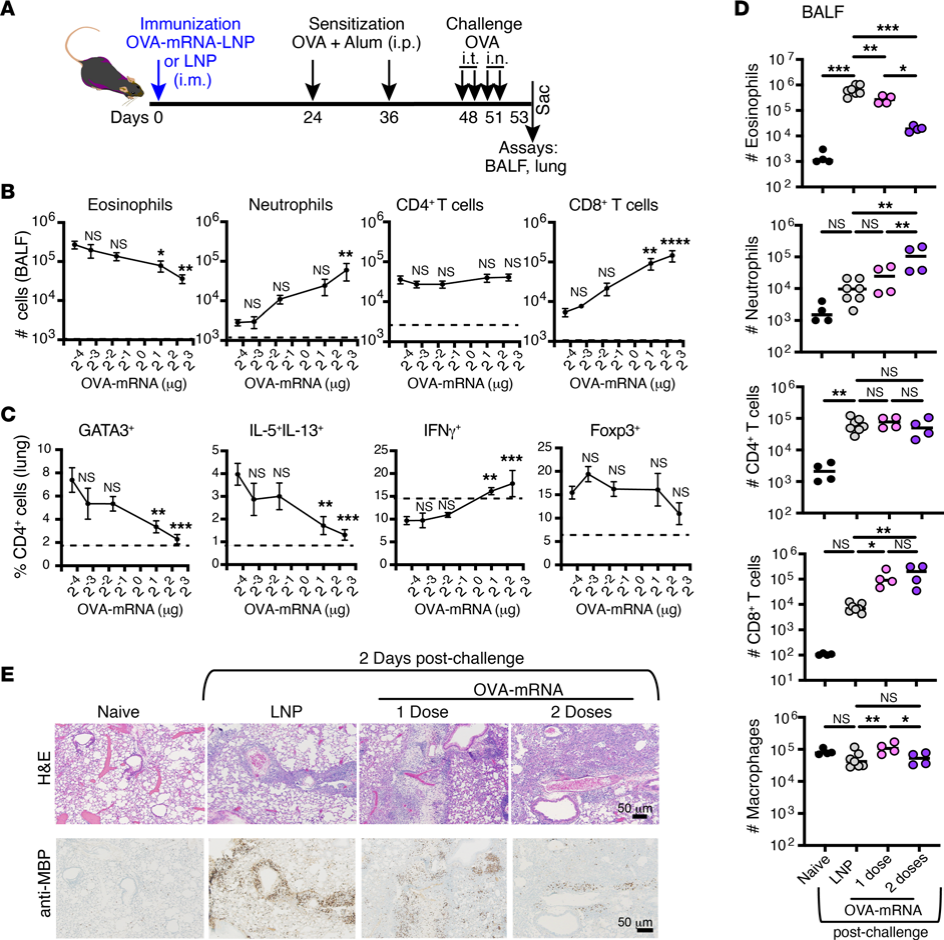
OVA-mRNA-LNP immunization reduces airway allergic responses in a dose-dependent manner. (**A**) Experimental workflow. Mice were subjected to a single intramuscular (i.m.) injection of different doses of OVA-mRNA-LNP (OVA-mRNA): 0, 0.1, 0.3, 2, and 5 μg. Sensitization with OVA+Alum was administrated intraperitoneally (i.p.) at days 24 and 36. Subsequently, daily challenges with 50 μg of OVA for 4 sequential days were applied to induce an allergic asthma response, 2 intratracheal (i.t.; days 48 and 49) followed by 2 intranasal (i.n.; days 50 and 51) injections. Bronchoalveolar lavage fluid (BALF) and lungs were collected on day 2 after the final challenge (day 53). (**B**) Quantification of cells in the BALF. (**C**) The frequency of GATA3^+^, Foxp3^+^, and cytokine-producing cells among CD4^+^ T cells in the lungs. (**B** and **C**) Dashed lines indicate cell values in naive mice without immunization and asthma induction. Shown is one of the 3 replicated experiments (*n* = 5–6); data are presented as mean ± SEM. (**D** and **E**) Mice received either 1 or 2 doses of the OVA-mRNA-LNP or empty LNP (LNP) administered 1 week apart (2 μg per injection) followed by a sensitization and airway challenge protocol (as in **A**). (**D**) Quantification of cells in the BALF (*n* = 4–7). Data are presented as the mean, with each circle representing an individual sample. Shown is 1 of the 3 replicated experiments. (**E**) H&E and anti–major basic protein (anti-MBP; eosinophils) staining of the lungs. Shown are representative panels at the same magnification. Scale bars: 50 μm. *P ≤ 0.05, **P ≤ 0.01, ***P ≤ 0.001, ****P ≤ 0.0001 by 1-way ANOVA with Tukey correction

To evaluate the impact of the vaccine under allergen-induced disease conditions, mice were subsequently given different doses of OVA-mRNA-LNP, ranging from 0 to 5 μg, sensitized with OVA protein mixed with alum adjuvant (OVA+Alum), and then challenged with OVA protein via intratracheal (i.t.) and intranasal (i.n.) routes (Figure 1A). Notably, mice receiving a higher dose of the OVA-mRNA-LNP exhibited a substantial reduction in the eosinophil count in the bronchoalveolar lavage fluid (BALF) (Figure 1B) and a decreased percentage of GATA3^+^ and IL-5^+^IL-13^+^ CD4^+^ T cells in the lung upon OVA challenge (Figure 1C), indicating a dose-dependent effect of the OVA-mRNA-LNP against Th2 responses in the airways. Concurrently, the number of CD8^+^ T cells and neutrophils in BALF, as well as the frequency of IFN-γ^+^ CD4^+^ T cells in the lungs, increased in an OVA-mRNA-LNP dose–dependent manner (Figure 1, B and C). The number of CD4^+^ T cells in BALF and the percentage of Foxp3^+^ Tregs in the lungs did not show marked changes.

To further improve the anti-allergic outcomes, we administered a booster dose of OVA-mRNA-LNP a week after the first immunization, followed by OVA+Alum sensitization and airway challenge with OVA protein (Supplemental Figure 3A). Mice given 2 doses of the OVA-mRNA-LNP exhibited a pronounced reduction in eosinophils in the BALF compared with those given a single dose, as indicated by flow cytometry analyses (Figure 1D, cell number, and Supplemental Figure 3B, cell frequency). This finding was substantiated through histologic staining of lung tissue using hematoxylin and eosin (H&E) and anti–major basic protein (anti-MBP) staining to detect eosinophils (Figure 1E). While a modest elevation in eosinophils was detected in the BALF (Figure 1D) and lung tissue (Figure 1E) of mice pretreated with 2 doses of OVA-mRNA-LNP in response to allergen administration (compared with naive mice), eosinophil levels remained nearly 100-fold lower than those in the LNP group, aligning with the decrease in Th2 cell responses observed in the lung. Although the proportion of CD4^+^ T cells was constant between mice receiving 1 or 2 doses of the OVA-mRNA-LNP (Figure 1D), 2 doses led to a more notable decline in CD4^+^ GATA3^+^ T cell frequency in the lungs upon OVA challenge, alongside an increase in CD4^+^ T cells producing IFN-γ, reflecting intensified Th1 responses (Supplemental Figure 3C). Additionally, neutrophils and CD8^+^ T cells were enriched in the BALF (Figure 1D and Supplemental Figure 3B), with higher frequencies of CCR5^hi^ and perforin^+^ CD8^+^ T cells in the lungs (Supplemental Figure 3D), underscoring enhanced CD8^+^ T cell migration and cytotoxic potential. Histologic examination corroborated the presence of accumulated lymphoid cells within the lung tissue of allergen-challenged, OVA-mRNA-LNP–immunized (single dose) and boosted (second dose) mice (Figure 1E). Taken together, these results demonstrate that immunization with the modified allergen-specific mRNA-LNP reduces Th2 effectors and increases Th1 and cytotoxic CD8 responses after allergen challenge.

### Clinical effects of allergen-specific mRNA-LNP vaccination in acute and chronic asthma.

**Figure 2:**
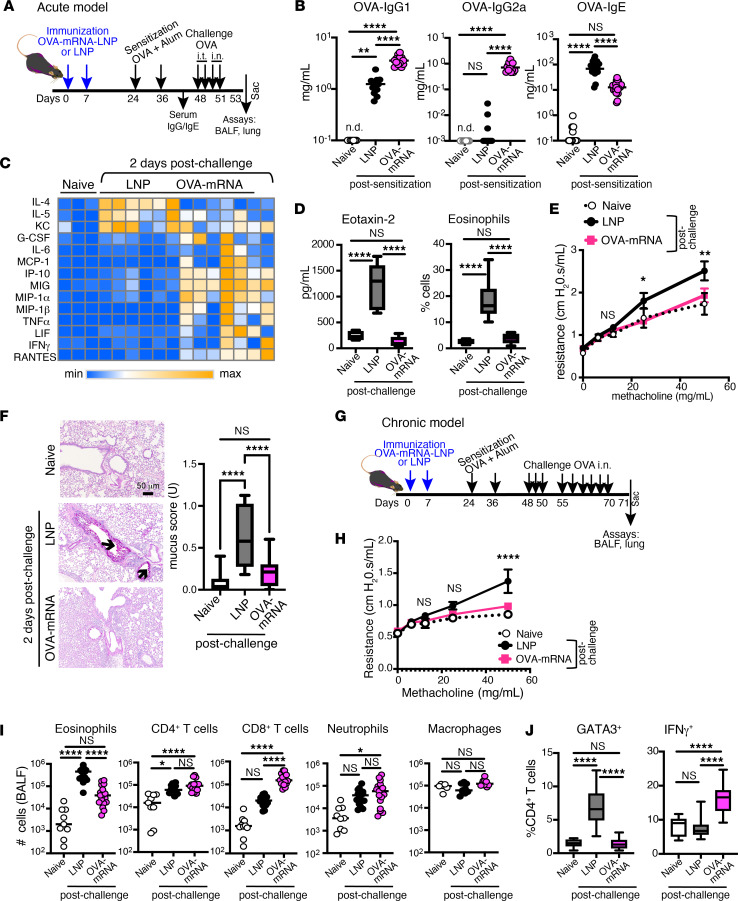
Clinical outcomes of allergen-specific mRNA-LNP vaccination in acute and chronic asthma models. (**A**) Experimental design. Mice received 2 doses of empty LNP (LNP) or OVA-mRNA-LNP (OVA-mRNA) on days 0 and 7. Mice were OVA+Alum–sensitized on days 24 and 36, and then OVA-challenged i.t. (days 48–49) and i.n. (days 50–51). Mice were sacrificed on day 53. Naive mice served as unmanipulated controls. (**B**) OVA-specific antibody levels were analyzed 1 week after the second sensitization. Data are pooled from 4 independent experiments (*n* = 16–20). Data represent individual values, with the mean per group; n.d., not detected. (**C**) Heatmap of cytokine and chemokine expression in the BALF. Values are normalized per row. (**D**) Eotaxin-2 levels (ELISA) in BALF (*n* = 4–10) and eosinophil frequencies in lung tissue (*n* = 8–17). (**E**) Airway resistance in response to increasing methacholine doses. Data are pooled from 3 independent experiments (*n* = 7–12). (**F**) Representative PAS-stained lung sections show mucus production; arrows indicate mucin-producing goblet cells. Mucus scores were quantified from 4 independent experiments (*n* = 9–26). Scale bar: 50 μm. (**D** and **F**) Box-and-whisker plots. (**G**) Experimental design. Mice received LNP or OVA-mRNA-LNP on days 0 and 7 and were sensitized on days 24 and 36 and challenged with 9 i.n. OVA doses. Mice were sacrificed on day 71. Naive mice served as unmanipulated controls. (**H**) Airway resistance was assessed 2 days after the last challenge (*n* = 4); data are mean ± SEM. Shown is 1 of the 2 replicated experiments. (**I**) BALF cell counts (*n* = 9–16). Each dot represents an individual mouse, with group mean shown. (**J**) Frequencies of lung CD4^+^ T cell subsets. Box-and-whisker plots summarize findings from 3 independent experiments (*n* = 9–16). *P ≤ 0.05, **P ≤ 0.01, ****P ≤ 0.0001 by 1-way or 2-way ANOVA with Tukey correction.

We aimed to extend the OVA-mRNA-LNP protective readouts to include clinically relevant features in acute and chronic asthma models. In humans, IgE typically mediates allergen-induced symptoms, whereas anti-allergen IgGs provide protection through a blocking mechanism (41, 42). Accordingly, allergen-specific antibody titers were measured after OVA-mRNA-LNP immunization and allergen sensitization of mice (Figure 2A). The OVA-mRNA-LNP–immunized group evidenced strong anti-OVA IgG1 and IgG2 responses, whereas the OVA-mRNA-LNP group compared with the LNP group had markedly reduced anti-OVA IgE upon OVA sensitization (Figure 2B). These findings provide early proof of concept for the potential efficacy of allergen-specific mRNA-LNP vaccination against IgE-mediated responses.

We next evaluated the cytokine profile in the BALF of airway allergen–challenged mice (Figure 2A). OVA-mRNA-LNP immunization markedly decreased Th2 cytokines, specifically IL-4 and IL-5 (Figure 2C), and the chemokine eotaxin-2 (CCL24), whose level correlated with the frequencies of eosinophils in the lung (Figure 2D). In contrast, factors associated with Th1-skewed responses, such as MIP-1α/β, MIG, IP-10, RANTES, TNF-α, and IFN-γ, were elevated in the OVA-mRNA-LNP compared with the LNP group (Figure 2C, Supplemental Figure 4A, and Supplemental Table 1). These results were substantiated by quantitative PCR analysis of lung tissue, which showed downregulation of *Il4*, *Il5*, *Il13*, *Ccl11*, and *Ccl24* and upregulation of *Ifng*, *Cxcl9*, *Cxcl10*, and *Ccl5* in the OVA-mRNA-LNP group (Supplemental Figure 4B), highlighting the anti-allergic effects of allergen-specific mRNA-LNP immunizations.

During the acute phase of experimental asthma (Figure 2A), OVA-mRNA-LNP–immunized mice were protected from developing allergen-induced airway hyperresponsiveness following OVA challenge, as shown by alleviated airway resistance to the inhaled bronchoconstrictor methacholine (Figure 2E), and exhibited reduced lung mucus production, as demonstrated by periodic acid–Schiff (PAS) staining (Figure 2F), compared with the increased airway resistance and mucus secretion observed in LNP-treated mice. The protective effects of OVA-mRNA-LNP immunization were also evident in a chronic asthma model with prolonged OVA exposure (Figure 2G). OVA-mRNA-LNP–treated mice displayed reduced airway resistance (Figure 2H), decreased eosinophilia (Figure 2I), diminished proportion of Th2 (GATA3^+^) cells, and increased IFN-γ^+^ CD4^+^ T cell frequency (Figure 2J), paralleling the acute response. Although CD8^+^ T cell counts were also elevated in the chronic model, neutrophil numbers remained low and comparable to those of LNP-treated mice (Figure 2I). Collectively, these results indicate that the allergen-specific mRNA-LNP provides protection against clinical features of experimental asthma in both acute and chronic stages.

### Allergen-specific mRNA-LNP pretreatment shifts the immune response from Th2 to Th1 in asthma.

To elucidate the mechanism behind allergen-specific mRNA-LNP vaccination, single-cell RNA sequencing (scRNA-Seq) was conducted on lung samples from naive mice and those pretreated with LNP or OVA-mRNA-LNP, followed by OVA+Alum sensitization and acute OVA airway challenge (Figure 2A). Uniform manifold approximation and projection (UMAP) clustering identified 15 distinct lung cell subpopulations with canonical markers for each subset (Supplemental Figure 5, A and B) (43–47). Transcriptomic analysis revealed that OVA-mRNA-LNP pretreatment reprograms both innate and adaptive immune responses, creating a distinct immunological landscape during allergen challenge. Among innate cells, the most prominent transcriptomic differences between LNP (asthmatic) and OVA-mRNA-LNP allergen-challenged groups were observed in neutrophils, interstitial macrophages, NK cells, dendritic cells, and plasma cells (Supplemental Tables 2–6). Pathway analysis revealed modulation of immune-regulatory pathways, including a shift toward Th1 polarization (e.g., IFN-γ regulation), regulation of adaptive activation through costimulatory checkpoints, and antigen presentation, as well as changes in cellular metabolism (Supplemental Figure 6A). These alterations collectively indicate a less permissive environment for Th2-driven allergic inflammation and a coordinated shift away from classical allergic responses, at the level of innate immunity.

**Figure 3:**
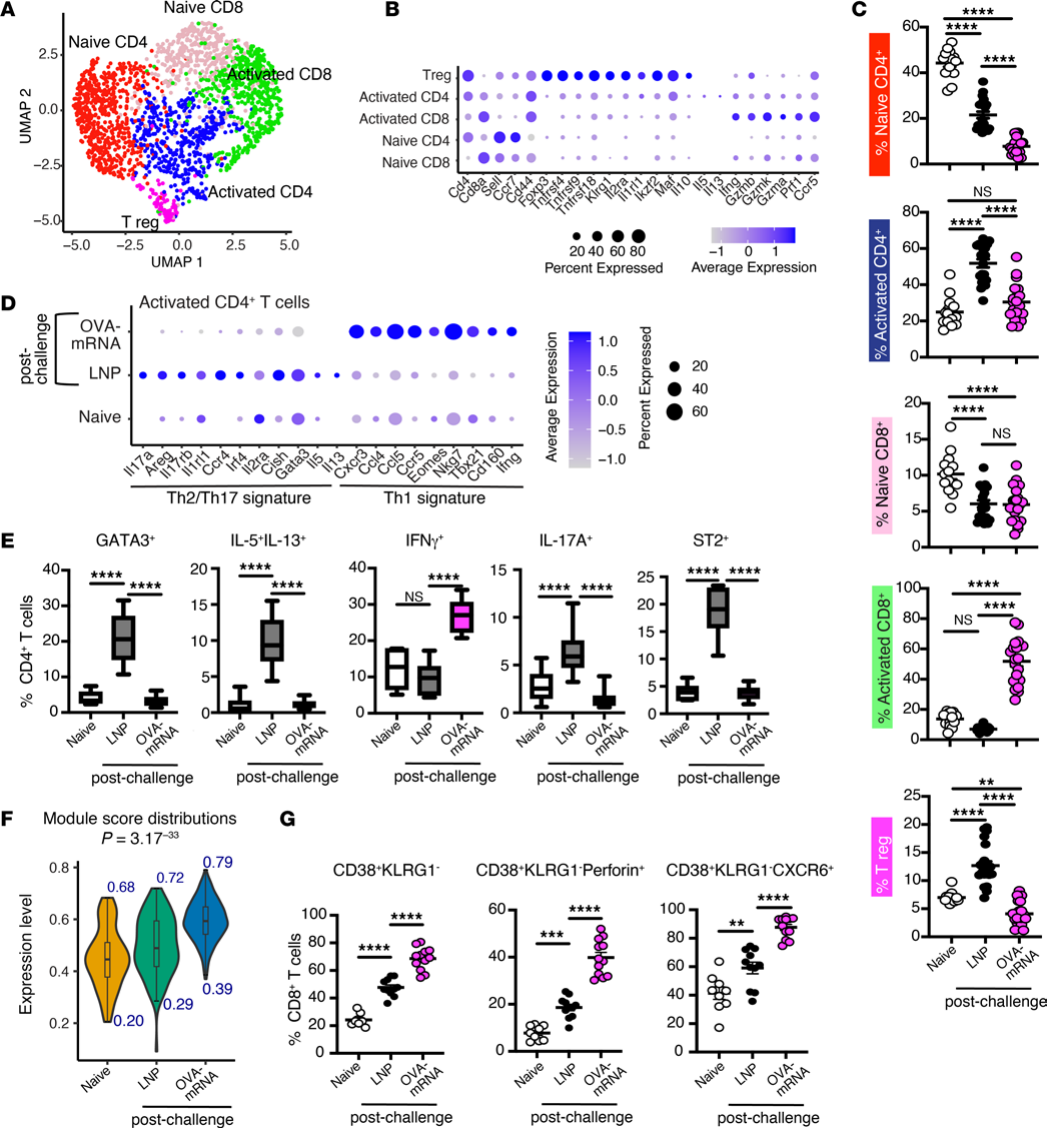
Allergen-specific mRNA-LNP immunization alters T cell responses in the lung during the allergen challenge. Lung tissue was harvested 2 days after the final challenge and subjected to Rhapsody single-cell RNA sequencing (**A**, **B**, **D**, and **F**) or flow cytometry (**C**, **E**, and **G**). Naive indicates unmanipulated mice. (**A**) UMAP of T cells from the lung tissue illustrates 5 cell clusters identified by unsupervised clustering, encompassing 3 different conditions (naive, LNP, and OVA-mRNA-LNP). (**B**) Selected marker gene expression in different T cell populations. (**C**) Flow cytometry analysis of T cell populations in the lungs. Foxp3^–^CD4^+^ or CD8^+^ cells were distinguished as naive (CD44^–^) or activated (CD44^+^) cells. Data pooled from 4 independent experiments are shown (*n* = 14–21). Data represent individual values, and the line represents the mean per group. (**D**) Expression of differentially expressed genes in activated CD4^+^ T cells per condition (*P* < 0.05 with fold change 2). (**E**) Frequencies of lung CD4^+^ T cells stained with indicated markers. (**F**) Module scores were calculated on a single-cell level for activated CD8^+^ T cells using 197 genes upregulated in human CD8^+^ T cells following SARS-CoV-2 mRNA immunization (49). The graph illustrates differences in the average expression levels for the specified gene set across cells from the naive, LNP, and OVA-mRNA-LNP samples (*P* = 3.17 × 10^–33^, ANOVA). (**G**) Frequencies of indicated CD8^+^ T cells in the lungs. Data represent individual values, and the line represents the mean per group. (**E** and **G**) Pooled data from 3 independent experiments are shown (*n* = 6–12). **P ≤ 0.01, ***P ≤ 0.001, ****P ≤ 0.0001 by 1-way ANOVA with Tukey correction.

Among αβ T cells, 5 subpopulations, including naive CD4^+^, naive CD8^+^, activated CD4^+^, activated CD8^+^ T cells, and Tregs, were identified by an extended panel of cell markers (Figure 3, A and B). Both scRNA-Seq (Supplemental Figure 6B) and flow cytometry analysis (Figure 3C) showed reduced frequencies of naive CD4^+^ and CD8^+^ T cells in allergen-challenged LNP- and OVA-mRNA-LNP–treated mice compared with naive mice. Activated CD8^+^ T cells were enriched in the allergen-challenged OVA-mRNA-LNP group, and activated CD4^+^ cells and Tregs were elevated in the LNP group (Figure 3C).

Further transcriptional analysis of activated CD4^+^ T cells revealed a Th2/Th17 signature in LNP-treated (asthmatic) mice and a distinct Th1 signature in OVA-mRNA-LNP–treated mice (Figure 3D). These differentially expressed genes included key lineage-specific transcriptional factors (*Irf4*, *Gata3*, *Eomes*, *Tbx21*), receptors (*Il17rb*, *Il1rl1*, *Ccr4*, *Il2ra*, *Cxcr3*, *Ccr5*, *Cd160*), functional molecules (*Areg*, *Cish*, *Nkg7*), and cytokines (*Il17a*, *Il5*, *Il13*, *Ifng*, *Ccl4*, *Ccl5*). Flow cytometry analysis corroborated these findings, showing a reduction of IL-5^+^IL-13^+^, GATA3^+^, IL-17A^+^, and ST2^+^ CD4^+^ T cells and elevation of IFN-γ^+^ CD4^+^ T cells in the OVA-mRNA-LNP group compared with the LNP group (Figure 3E).

### Pretreatment with allergen-specific mRNA-LNP vaccine and mTOR inhibitor modulates CD8^+^ T cell responses in experimental asthma.

We evaluated the transcriptional profile of activated CD8^+^ T cells by calculating module scores (48) based on the gene signature of human CD8^+^ T cells recently identified as the most potent responders following SARS-CoV-2 mRNA vaccination (49). This human gene set included 197 genes and was enriched with genes related to cytotoxic and effector functions of T cells, TCR signaling, antigen processing, and metabolism (Supplemental Table 7) (49). Notably, activated CD8^+^ T cells from the OVA-mRNA-LNP group exhibited, on average, higher module score values compared with those of the naive and LNP groups, indicating close resemblance to the human CD8^+^ T cells induced by SARS-CoV-2 mRNA vaccination (Figure 3F). Furthermore, flow cytometry analysis revealed that, similar to human CD8^+^ T cells induced by the SARS-CoV-2 mRNA vaccine (49), the allergen-challenged OVA-mRNA-LNP group showed an elevated frequency of CD38^+^KLRG1^–^ CD8^+^ T cells, which demonstrated enhanced perforin production and expressed the activation marker CXCR6 (Figure 3G). These results demonstrate that the CD8^+^ T cell response following allergen-specific mRNA-LNP vaccination is conserved at least in part across species and vaccine immunogens.

**Figure 4:**
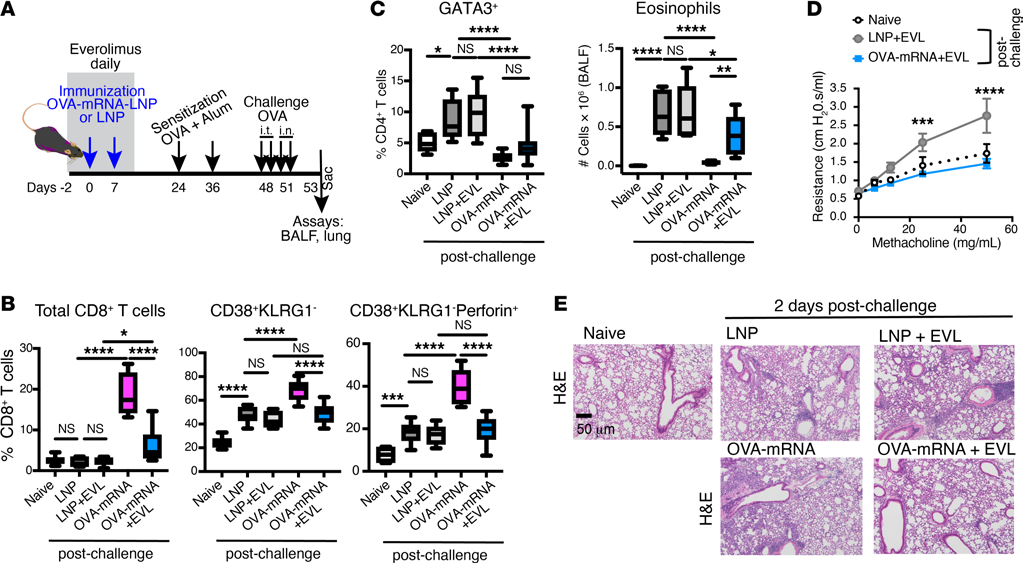
OVA-mRNA-LNP in combination with mTOR inhibitor reduces inflammation and frequencies of cytotoxic CD8^+^ T cells in lungs. (**A**) Experimental design. Mice received 2 doses of empty LNP (LNP) or OVA-mRNA-LNP vaccine (OVA-mRNA) on days 0 and 7 (blue arrows). Everolimus (EVL) was administered daily from day –2 to day 12 (shaded area). Mice were OVA+Alum–sensitized on days 24 and 36 and subsequently OVA-challenged for 4 consecutive days (days 48–51), 2 i.t. and then 2 i.n. BALF and lung tissue were collected 2 days after the final challenge (day 53). Naive mice served as unmanipulated controls. (**B**) Frequencies of indicated CD8^+^ T cells in the lungs. (**C**) Frequencies of CD4^+^ cells expressing GATA3 transcription factor in the lungs, and eosinophil count in the BALF. (**B** and **C**) Box-and-whisker plots. (**D**) Analysis of the airway resistance in the indicated mice in response to increasing concentrations of methacholine. Data are pooled from 2 independent experiments (*n* = 6–10). Data are mean ± SEM. (**B**–**D**) Graphs depict data from combined experiments (*n* = 9–12). (**E**) H&E staining of the lungs. Shown are representative panels for each group at the same magnification. Scale bar: 50 μm. *P ≤ 0.05, **P ≤ 0.01, ***P ≤ 0.001, ****P ≤ 0.0001 by 1-way or 2-way ANOVA with Tukey correction.

Although CD8^+^ T cells may exert anti-allergic effects (50), their cytotoxic functions may be undesirable and could contribute to non-allergic inflammation. Previous studies suggest that mTOR inhibitors suppress CD8^+^ T cell effector function when coupled with a potent antigenic signal (51, 52). Therefore, we hypothesized that coadministering the OVA-mRNA-LNP with the mTOR inhibitor everolimus may modulate CD8^+^ T cell cytotoxic activity in the allergic model (Figure 4A). Indeed, mTOR inhibition attenuated OVA-mRNA-LNP–induced CD8^+^ T cells in the lung and the CD38^+^KLRG1^–^ CD8^+^ T cell subset, including perforin production (Figure 4B), while preserving the anti-allergy effect of the vaccination. This was evidenced by decreased frequencies of Th2 cells in the lungs, lower eosinophil counts in the BALF (Figure 4C), normalized airway hyperresponsiveness (Figure 4D), and reduced inflammatory infiltration in lung tissues (Figure 4E).

### The anti-allergic potential of Der p1–mRNA–LNP.

**Figure 5:**
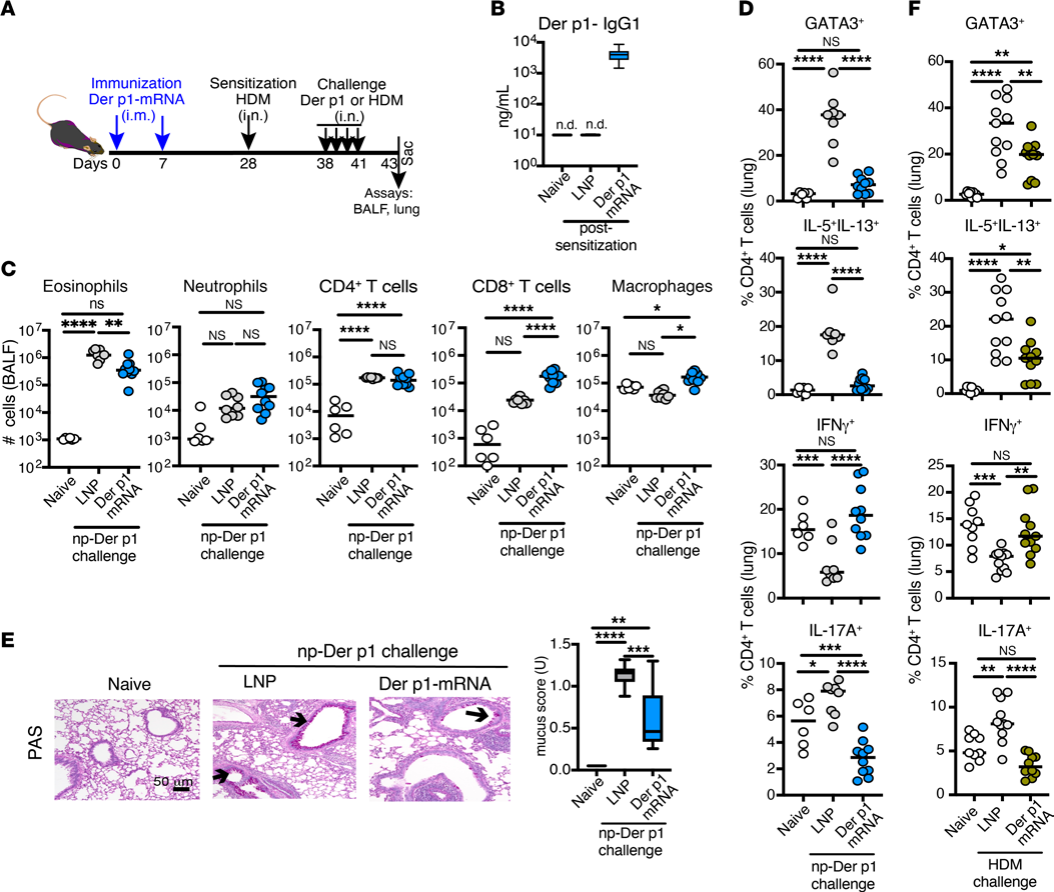
Der p1–mRNA–LNP protects against allergic responses to HDM. (**A**) Experimental design. Mice were injected with LNP or Der p1–mRNA–LNP on days 0 and 7 (**B**–**F**); both groups were sensitized with HDM (day 28) (**C**–**F**) and challenged i.n. with naturally purified Der p1 (np-Der p1) protein (**C**–**E**) or HDM extract (**F**) (days 38–41). (**B**) Box-and-whisker plot of Der p1–IgG1 antibody levels in the serum of naive, LNP, or Der p1–mRNA–LNP–vaccinated mice on day 26 (*n* = 9–20). (**C**) Quantification of cells in the BALF on day 2 after the last challenge. (**D** and **F**) The frequency of GATA3^+^ and cytokine-producing cells among CD4^+^ T cells in the lungs. (**E**) Representative PAS-stained sections in bronchi and bronchiole for mucus production. Arrows indicate goblet cells producing mucin. The box-and-whisker plot shows a mucus score pooled from 2 independent experiments (*n* = 6–8). Scale bar: 50 μm. (**C**, **D**, and **F**) Data are pooled from 2 or 3 independent experiments (*n* = 6–12), the dots represent individual mice, and the line represents the mean per group. *P ≤ 0.05, **P ≤ 0.01, ***P ≤ 0.001, ****P ≤ 0.0001 by 1-way ANOVA with Tukey correction. n.d., not detected.

To better approximate physiological conditions, we evaluated the impact of allergen-specific mRNA-LNP vaccination on the immune response to house dust mite (HDM). Mice were immunized with an allergen-specific mRNA-LNP encoding the major HDM allergen Der p1, followed by sensitization with HDM and challenges with naturally purified Der p1 (np-Der p1) protein or HDM extract (Figure 5A). Der p1–mRNA–LNP vaccination increased production of Der p1–specific IgG1 antibodies (Figure 5B), indicating active immunogenicity. Following challenge with np-Der p1 protein, immunized mice exhibited reduced allergic responses, including lessened eosinophilia, decreased Th2 (GATA3^+^ and IL-5^+^IL-13^+^) and Th17 (IL-17A^+^) cell frequencies, and lowered mucus production (Figure 5, C–E). Additionally, elevated CD8^+^ T cell frequency and enhanced IFN-γ production by CD4^+^ T cells were observed in Der p1–mRNA–LNP–vaccinated mice compared with LNP-treated mice (Figure 5, C and D). A reduction in Th2 and Th17 cell populations, along with an increase in IFN-γ–producing cells, was also detected in Der p1–mRNA–LNP mice challenged with HDM extract (Figure 5F), although the anti-allergic effect was not complete (Supplemental Figure 7). These results highlight the potential of the Der p1–mRNA–LNP vaccines to counteract HDM-induced allergic inflammation, warranting further research to optimize their efficacy and broader applications.

### Therapeutic efficacy of allergen-specific mRNA-LNP on established atopy.

**Figure 6:**
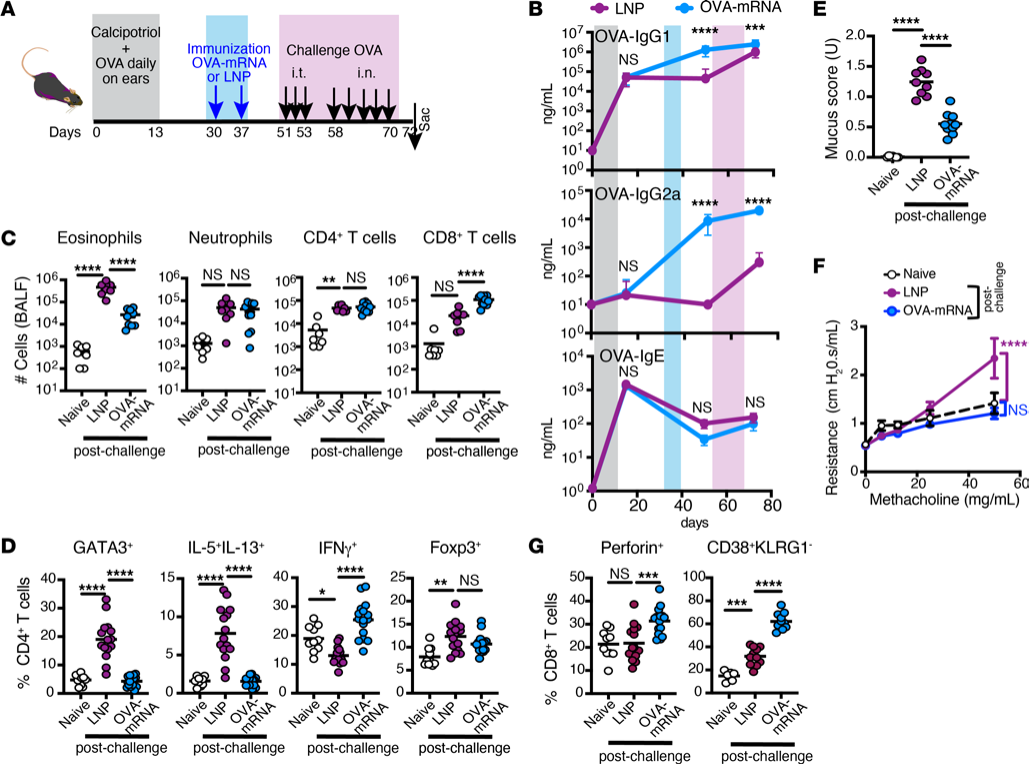
Treatment with allergen-specific mRNA-LNP mitigates asthma responses in mice. (**A**) Experimental design. Mice were cutaneously sensitized (gray area) with OVA in the presence of calcipotriol (MC903) daily in the ears for 2 weeks. Immunization (blue area) with LNP or OVA-mRNA-LNP was provided i.m. on days 30 and 37 (blue arrows), followed by 8 airway challenges (pink area and black arrows) with OVA i.t. (daily between day 51 and day 53) and then i.n. (day 58 through day 70). Mice were sacrificed 2 days after last challenge. Naive mice were used as unmanipulated controls. (**B**) Kinetics of OVA-IgG1, OVA-IgG2a, and OVA-IgE antibody secretion in serum of mice treated with LNP or OVA-mRNA-LNP (*n* = 16–20). Colored areas indicate treatments as in **A**. (**C**) The graphs show quantification of cells in the BALF pooled from 2 independent experiments (*n* = 7–10). (**D**) The frequency of GATA3^+^, Foxp3^+^, and cytokine-producing cells among CD4^+^ T cells. (**E**) A mucus score based on the percentage of PAS staining in the bronchi (*n* = 9–10). (**F**) Analysis of the airway resistance in the indicated mice in response to increasing concentrations of methacholine. Data are pooled from 2 independent experiments (*n* = 6–11). (**G**) The frequency of perforin^+^ and CD38^+^KLRG1^–^ cells among CD8^+^ T cells in the lungs. (**D**, **E**, and **G**) Data are pooled from 3 independent experiments (*n* = 10–16). (**B** and **F**) Data are mean ± SEM. (**C**–**E** and **G**) The dots represent individual mice, and the line represents the mean per group. *P ≤ 0.05, **P ≤ 0.01, ***P ≤ 0.001, ****P ≤ 0.0001 by 1-way or 2-way ANOVA with Tukey correction.

To investigate the therapeutic potential of the allergen-specific mRNA-LNP as an immunotherapy for established allergies, mice were sensitized by application of the allergen to the skin in the presence of a vitamin D analog, calcipotriol (MC903), which mimics the atopic dermatitis condition (53). This sensitization was followed by i.m. treatment with either OVA-mRNA-LNP or empty LNP and subsequent repeated airway challenges with OVA (Figure 6A). Sensitization with OVA+MC903 resulted in an increased production of OVA-specific IgE and IgG1, but not IgG2a, antibodies (Figure 6B, day 15). Both OVA-specific IgG1 and IgG2a levels were remarkably amplified following treatment with OVA-mRNA-LNP and remained elevated beyond levels seen following treatment with LNP, even after the OVA challenges (Figure 6B, day 72). The IgE level decreased after sensitization (day 50) regardless of OVA-mRNA-LNP treatment, indicating that mRNA does not worsen humoral allergic responses in allergen-challenged mice. Upon repeated OVA airway challenges, OVA-mRNA-LNP–treated mice exhibited notable reductions in the asthma-related features, including eosinophilia, Th2 response, mucus secretion, and airway hypersensitivity (Figure 6, C–F). Additionally, these mice demonstrated increased frequencies of IFN-γ^+^ CD4^+^ T cells and perforin^+^ CD8^+^ T cells expressing the CD38^+^KLRG1^–^ phenotype (Figure 6G), as was observed in the prophylaxis model (Figure 3G). In contrast, LNP-treated mice developed lung eosinophilia (Figure 6C), a rise in Th2 cells (Figure 6D), excessive mucus production in the tissue (Figure 6E), and heightened airway resistance to methacholine (Figure 6F). Notably, OVA-mRNA-LNP–treated mice showed no signs of anaphylaxis, either immediately or at any point during the hours or days following OVA-mRNA-LNP injections (Supplemental Figure 8). In contrast, in a parallel cohort of mice, subcutaneous administration of OVA (54) was associated with a high rate of systemic adverse events as manifested by a severe drop in body temperature following the first dose of OVA immunotherapy (Supplemental Figure 8, OVA s.c.). Collectively, these findings underscore the potential safety and efficacy of antigen-specific mRNA-LNP to treat allergic inflammation and asthma symptoms.

## Discussion

With the rising prevalence of allergic diseases, affecting up to 80% of the population in certain regions of the world (1–3, 55), there is a need to focus on better treatment and prevention strategies (56). Accordingly, a number of promising approaches are under way, including subcutaneous and sublingual administration of allergen extracts, microbial derivative adjuvants, recombinant allergens including hypoallergenic variants, epitope-based immunotherapies, allergen-encoding nucleic acids, and advanced delivery systems (7, 8, 12, 15, 18, 57–59). Here, we demonstrate effectiveness of allergen-specific, nucleoside-modified mRNA-LNP vaccination in preventing and treating type 2 immunity in mice by modulating T cell differentiation, mirroring effects seen in mRNA vaccines encoding viral antigens (21, 37, 38). Immunization with allergen-specific mRNA-LNP as a preventive strategy enhanced Th1 cell generation, effectively counteracting pro-allergic Th2 responses in both acute and chronic allergy models. Additionally, the finding that allergen-specific mRNA-LNP also abrogates the Th17 response widens the potential scope of mRNA-LNP vaccination to Th17-associated asthma, such as glucocorticoid–resistant asthma, and other inflammatory diseases such as colitis (60–62). In both preventive and therapeutic models, allergen-specific mRNA-LNP immunization modulates B cell polarization, generating avid humoral immunity characterized by antigen-specific IgG1 and IgG2a. As a result, tissue eosinophilia was substantially decreased, mucus production was diminished, and airway hypersensitivity was normalized. The ability of the allergen-specific mRNA-LNP to induce high levels of anti-allergen IgG isotypes, which likely include blocking antibodies, and reduce allergen-specific IgE in a preventive model underscores the potential utility of this approach in treating antibody-dependent allergic diseases, including food allergy, a subject that deserves attention. A notable finding was the ability of allergen-specific mRNA-LNP to elicit a CD8^+^ T cell response in mice that resembled that seen following SARS-CoV-2 mRNA vaccination in humans (49). This conserved immune response across species and despite the diverse type of antigens encoded by the mRNA underscores the versatility of the mRNA-LNP technology for a variety of medical applications. Though the induction of CD8^+^ T cells by the allergen-specific mRNA-LNP vaccination has not proven to be detrimental in our experimental systems, we were able to attenuate the response by coadministration of an mTOR inhibitor with the allergen-specific mRNA-LNP, demonstrating the adaptability of this approach. The finding that mTOR inhibition modifies the response to mRNA-LNP vaccination has potential broad implications and deserves further attention.

The potent and rapid anti-allergic immunity uncovered in our preclinical studies, combined with the beneficial features of the mRNA-LNP platform in humans — including overall safety, cost-effectiveness, and rapid deployment — highlights its potential as a promising clinical approach (28, 31). Notably, although Der p1–mRNA–LNP immunization attenuated HDM-induced Th2 responses and enhanced frequency of CD8^+^ T cells, it was unable to reduce eosinophilia. This suggests that targeting a single allergen may be insufficient to fully mitigate the complex immune responses elicited by multi-component allergens such as HDM. Additionally, the intrinsic properties of the encoded allergen should be considered in the design of mRNA-based vaccines. A recent study comparing native Der p1 with a hypoallergenic Der p1_C132A_ variant in liver-targeted protein-based immunotherapy revealed substantial differences in immunomodulatory efficacy (63). These findings indicate that both the number and nature of allergen targets are critical parameters in the development of effective anti-allergic mRNA-LNP vaccines. It is noteworthy that mRNA-LNP formulation can be engineered to express multiple proteins and encode their hypoallergic variants (23, 64), which provides an opportunity to incorporate multiple allergenic proteins into a single treatment. These strategies may be applied to address not only airway inflammation, but also other allergic disease involving adaptive immune responses, such as food allergy, atopic dermatitis, allergic rhinitis, and more.

## Methods

### Sex as a biological variable.

Both female and male mice aged 6–12 weeks were included in this study, and similar findings are reported for both sexes.

### Animals.

All mouse strains used were on the C57BL/6 genetic background (B6J, 000664) and were purchased from The Jackson Laboratory. OTII mice (004194) were bred with CD45.1 mice (002014). Mice were bred and housed under specific pathogen–free conditions. All experiments complied with protocols approved by the Cincinnati Children’s Hospital Medical Center (CCHMC) Animal Use and Care Committee.

### mRNA design and production.

The amino acid sequences of the OVA and Der p1 were obtained from GenBank accession numbers MF321513.1 and P08176.2, respectively. The sequences underwent codon optimization and GC enrichment using our proprietary algorithm to improve expression and reduce potential immunogenicity of the in vitro–transcribed mRNA. The codon-optimized allergen sequences were gene-synthesized by GenScript and cloned into an in vitro transcription template containing an optimized T7 promoter, 3′-untranslated region (UTR), 5′-UTR, and 100-adenine tail. The allergen nucleoside–modified mRNAs were prepared using the MegaScript Transcription Kit (Thermo Fisher Scientific), cotranscriptionally capped using the CleanCap system (TriLink Biotechnologies), purified using a modified cellulose base chromatography method (65), precipitated, eluted in nuclease-free water, and quantified using the NanoDrop One system (ThermoFisher Scientific). Length and integrity were determined using agarose gel electrophoresis. Endotoxin content was measured using the GenScript Toxisensor chromogenic assay, and values were below detection levels (0.1 EU/mL). mRNA was frozen at –20°C until formulation.

### LNP production and characterization.

Cellulose-purified RNAs containing *N*1-methylpseudouridine (m1Ψ) were encapsulated in LNPs using a self-assembly process as previously described wherein an ethanolic lipid mixture of ionizable cationic lipid, phosphatidylcholine, cholesterol, and polyethylene glycol–lipid was rapidly mixed with an aqueous solution containing mRNA at acidic pH (66). The LNP formulation used in this study is proprietary to Acuitas Therapeutics; the proprietary lipid and LNP compositions are described in US patent US10,221,127. Additional LNPs were formulated using SM-102, distearoylphosphatidylcholine cholesterol, and 1,2-dimyristoyl-rac-glycero-3-methoxypolyethylene glycol-2000 (BroadPharm) in a molar ratio of 50:10:38.5:1.5. Lipids were dissolved in ethanol and combined with aqueous mRNA with a flow rate ratio of 3:1 using a NanoAssemblr Ignite (Cytiva). mRNA-LNPs were diluted 40:1 in PBS containing 10% sucrose, concentrated to 1 mg/mL using centrifugal filters (MilliporeSigma), and stored at –80°C. The hydrodynamic size, polydispersity index, and ζ-potential of mRNA-LNPs were measured using a Zetasizer Nano ZS90 (Malvern Instruments). The mRNA encapsulation efficiency was determined using a modified Quant-iT RiboGreen RNA assay (Invitrogen). Particles had a size of 80 nm, a polydispersity index of 0.02, and an encapsulation efficiency above 95%.

### Adoptive transfer.

Cells were collected from LNs and spleens of OTII mice expressing CD45.1, forced through a 70 μm cell strainer, and treated with ammonium-chloride-potassium (ACK) lysing buffer to remove red blood cells. Naive CD4^+^ T cells were isolated using the EasySep Mouse Naive CD4^+^ T Cell Isolation Kit. A total of 3 × 10^6^ cells per mouse were intravenously injected in 200 μL PBS into sex-matched C57BL/6 recipients, which were immunized 3 days after cell injection. LNs were collected at the specified time points.

### OVA-mRNA-LNP, Der p1–mRNA–LNP, empty LNP, everolimus treatment, and endotoxin-free OVA administration.

In the preventive protocol, naive wild-type C57BL/6 mice were injected i.m. in the lower left leg with increasing doses (0.1, 0.3, 1, 2, and 5 μg) or a consistent amount of the m1Ψ-mRNA-LNPs encoding an ovalbumin protein (2 μg; OVA-mRNA-LNP), Der p1 protein (5 μg; Der p1–mRNA–LNP), or empty LNP on days 0 and 7, as indicated. Intraperitoneal (i.p.) treatment with everolimus (5 mg/kg of body weight) commenced 2 days before the first immunization and continued daily for 14 days.

In the treatment protocol, mice sensitized by MC903 (1 nmol in 25 μL per ear; Tocris) and OVA (50 μg in 5 μL per ear; MilliporeSigma) were injected with 10 μg of OVA-mRNA-LNP or empty LNP twice, with a 7-day interval between injections. For standard immunotherapy, mice were given 3 subcutaneous (s.c.) injections every third day with 1 mg of endotoxin-free OVA (Ovalbumin EndoFit, InVivoGen) in 100 μL endotoxin-free saline.

A complete list of reagents is provided in Supplemental Table 8.

### Sensitization and allergen challenge protocol for allergic asthma.

LNP- or OVA-mRNA-LNP–immunized mice were sensitized through i.p. injection with 100 μg OVA (MilliporeSigma) emulsified in 100 μL of Imject ALUM (Thermo Fisher Scientific) or aluminum hydroxide gel (InVivoGen) in a total volume of 200 μL per mouse on days 24 and 36 after first immunization. Subsequently, on days 48 and 49, mice were challenged i.t., followed by i.n. challenge on days 50 and 51 with 50 μg of OVA. In the chronic model, mice were challenged i.n. with OVA (50 μg in 30 μL) 3 times daily, followed by 6 additional challenges every other day.

In the HDM model, mice were sensitized i.n. with 80 μg of HDM extract (Greer Laboratories Inc.) in 40 μL. Ten days later, mice were challenged i.n. with HDM (10 μg in 30 μL) or np-Der p1 protein (10 μg in 30 μL; InBio) for 4 consecutive days.

In the treatment model, mice were sensitized with MC903 (1 nmol in 25 μL per ear; Tocris) plus OVA (50 μg in 5 μL per ear) applied to both ears daily for 14 days, followed by immunization with OVA-mRNA-LNP (10 μg, i.m.) or LNP 2 weeks later. Two weeks after the last immunization, mice were challenged i.t. with OVA (50 μg in 30 μL) 3 times daily, followed by 5 additional i.n. OVA challenges every other day.

Forty-eight hours after the final OVA or HDM challenge, mice were euthanized with pentobarbital and subsequently examined. Sex- and age-matched naive mice served as untreated controls.

### BALF and lung preparation.

For BALF collection, lungs were lavaged once with 0.8 mL of PBS through cannulation of the tracheal tube. Total cell numbers in the collected fluid were counted using a hemocytometer. BALF was centrifuged, and supernatant was stored at –80°C for enzyme-linked immunosorbent assay (ELISA) or cytokine/chemokine multiplex assays. Cells were resuspended in PBS with 2% fetal bovine serum (FBS) and subjected to live flow cytometry staining on the same day.

One lobe of the lungs was minced and subjected to Liberase TL digestion (0.25 mg/mL) in the presence of DNase I (0.5 mg/mL) at 37°C for 1 hour. The resulting suspension was forced through 70 μm cell strainers, washed, and resuspended in RPMI plus 10% FBS with DNase I (0.5 mg/mL). These cells were used for live or intracellular staining or subjected to scRNA-Seq or quantitative PCR.

### Lung histopathology.

Two days after the final OVA challenge, mice were euthanized with pentobarbital for histologic examination. Lungs were inflated through the tracheal tube with 0.7 mL of 10% neutralized buffered formalin, removed, fixed overnight in 10% formalin, and dehydrated in 70% ethanol. Lung tissues were embedded in paraffin and cut into 5-μm-thick sections, which were subsequently deparaffinized. The sections were stained with hematoxylin and eosin (H&E) or periodic acid–Schiff (PAS) or subjected to immunohistochemical stain against murine eosinophil major basic protein (MBP) as previously reported (67).

Mucus-containing goblet cells were detected by PAS staining. PAS-stained goblet cells in the airway epithelium were quantified using a scoring system (0: <5% goblet cells; 1: 5%–25%; 2: 25%–50%; 3: 50%–75%; 4: >75%), as previously described (68). Twenty to fifty airways per mouse were examined, and the average score was calculated. Histologic analyses were performed in a blinded manner by the same person.

### Airway hyperresponsiveness measurements.

The FlexiVent system (SCIREQ Scientific Respirator Equipment Inc.) was used to evaluate airway hyperresponsiveness 48 hours after final OVA exposure. Mice were anesthetized with a mixture of ketamine (90–120 mg/kg) and xylazine (10–20 mg/kg) and paralyzed with pancuronium bromide (0.8–1.2 mg/kg). Tracheae were cannulated with an 18-gauge blunt cannula. Mice were ventilated at 150 breaths/min and 3.0 cm water positive and expiratory pressure and allowed to stabilize on the machine for 2 minutes. Mice were then exposed to methacholine (0, 6.25, 12.5, 25, and 50 mg/mL) aerosolized in PBS for 15 seconds and ventilated for an additional 10 seconds. Ventilation cycle measurements were taken until resistance peaked. Airways were then re-recruited by deep inflation, and the next methacholine dose was administered. Analyses were performed in a blinded manner by the same person.

### Anaphylactic response.

To monitor anaphylaxis, the anal temperature was measured before the injection of endotoxin-free OVA or OVA-mRNA-LNP and subsequently every 10–15 minutes during the first hour after injection. Thereafter, mice were visually assessed hourly for 6 hours and then twice daily for 3 days.

### Flow cytometry staining.

Freshly obtained cells from BALF and lungs of naive or allergen-sensitized/challenged mice were stained for surface markers, including anti–mouse CD45, CD11c, CD64, CD11b, CD3, CD4, CD8, Gr1, or Ly6G, and Siglec-F, at 4°C for 60 minutes.

For surface and intracellular staining of T cells, cells from the lungs or LNs were incubated in PBS at 4°C for 10 minutes with the fixable viability dye eFluor 780. After washing with PBS containing 2% FBS, cells were fixed and permeabilized using the Foxp3/Transcription Factor Fixation/Permeabilization Kit according to the manufacturer’s instructions (Thermo Fisher Scientific). Subsequently, cells were stained with anti-mouse fluorescent antibody cocktails. For lungs, anti–mouse CD44, CD4, CD8, GATA3, Foxp3, KLRG1, CD38, CXCR6, ST2, and CCR5 antibodies were used (Supplemental Table 8). For LNs, anti–mouse CD44, CD4, CD45.1, CD45.2, GATA3, Foxp3, CD25, T-bet, Bcl6, and PD-1 antibodies were used. The staining process occurred at room temperature for 60 minutes.

For intracellular cytokine detection, cells from lungs or LNs were stimulated with phorbol 12,13-dibutyrate (500 ng/mL) and 1 μM ionomycin in the presence of brefeldin A in an incubator at 37°C for 4 hours. Cells were stained in PBS for 10 minutes with the fixable viability dye eFluor 780 in the presence of brefeldin A and then were fixed and permeabilized with the Foxp3/Transcription Factor Fixation/Permeabilization Kit. T cells were stained with fluorescent antibodies against mouse CD4, CD8, CD44, GATA3, Foxp3, IL-2, IL-4, IL-5, IL-13, IL-17A, IFN-γ, TNF-α, CCR5, KLRG1, CD38, and perforin.

All staining was performed in the presence of Fc Block (anti–mouse CD16/CD32, BD Biosciences). A complete list of reagents and antibodies is provided in Supplemental Table 8. Data acquired with a BD LSR Fortessa flow cytometer (BD Biosciences) were analyzed by FlowJo software (Tree Star Inc.). The absolute number of cells in each population in the BALF was calculated by multiplication of the total cell numbers in the collected BALF by the percentage of flow cytometry–gated cells.

### Quantitative PCR.

Mouse lungs were collected and homogenized in Trizol with QIAGEN beads. mRNA was isolated following the Trizol protocol and purified using the Quick-RNA Miniprep Kit (Zymo Research). For real-time PCR analysis, mRNA was reverse-transcribed with the ProtoScript cDNA Synthesis Kit (New England Biolabs). Primers specific for mouse *Il4*, *Il5*, *Il13*, *Ifng*, *Cxcl9*, *Cxcl10*, *Ccl5*, *Ccl11*, *Ccl24*, and *Eif3k* were obtained from Integrated DNA Technologies (Supplemental Table 9). SYBR Green Real-Time PCR was performed and analyzed using the ABI Quant-Studio 7 Flex Real-Time PCR system (Thermo Fisher Scientific). The transcripts of interest were normalized to *Eif3k* cDNA.

### Cytokine multiplex assay, CCL24 ELISA, and antibody titer determinations.

BALF was collected 2 days after the last challenge and analyzed with the Mouse Cytokine/32-Plex Discovery Assay (Eve Technologies Corp.). The Mouse CCL24/Eotaxin-2/MPIF-2 DuoSet ELISA Kit (R&D Systems) was used to measure the concentration of eotaxin-2 in the BALF.

To measure OVA-specific antibody secretion, blood was collected from the tail 2 weeks after the last dose of OVA-mRNA-LNP or Der p1–mRNA–LNP immunization, 7 days after the last OVA+Alum sensitization, or 1 day after OVA+MC903 sensitization. Serum was separated by centrifugation at 2,236*g* for 5 minutes at room temperature. Ninety-six-well ELISA plates were coated with 10 μg/mL of OVA overnight at 4°C. Serum samples were added in different dilutions. Detection of OVA-specific antibodies from serum was performed using horseradish peroxidase–conjugated (HRP-conjugated) anti-IgG1, anti-IgG2a, or biotinylated anti-IgG2b followed by HRP according to the manufacturer’s instructions. For substrate, 3,3′,5,5′-tetramethylbenzidine (TMB) was used. The colorimetric reaction was stopped with 10% H_3_PO_4_, and the optical density was quantified using an ELISA plate reader at 450 nm, with subtraction of background absorbance at 570 nm. OVA-specific monoclonal antibodies IgG1, IgG2a, and IgG2b were used as standards (Supplemental Table 8). To prevent nonspecific binding, SuperBlock Blocking Buffer (Thermo Fisher Scientific) was used for blocking and dilutions. For the detection of OVA-specific IgE in murine serum, an ELISA kit from BioLegend was used. For the detection of Der p1–specific IgG, an ELISA kit from Chondrex was used.

### scRNA-Seq collection.

Single-cell transcriptome analysis of the murine esophagus was performed using the BD Rhapsody Single-Cell Analysis System (BD Biosciences). To obtain a single-cell suspension, the left lobe of the lungs was minced and incubated with Liberase TL (0.25 mg/mL) in the presence of DNase I (0.5 mg/mL) for enzymatic digestion. Cells from 2 mice per group were pooled, treated with ACK lysing buffer for 1 minute, washed, passed through a 70 μm cell strainer, and counted using the TC20 Automated Cell Counter (Bio-Rad). Subsequently, cells were processed on the Rhapsody platform following the manufacturer’s protocol. Cells from each treatment group were labeled with individual sample tags (BD Mouse Single-Cell Multiplexing Kit, catalog 633793). Before loading into the cartridges, cells were quantified on the Rhapsody scanner after staining with Vybrant DyeCycle Green (Invitrogen, V35004) for 5 minutes at room temperature. Barcoded samples were pooled in equal amounts for each of the 3 treatment groups (naive, LNP, OVA-mRNA-LNP), and 60,000 cells were loaded into each cartridge, with 2 cartridges used for sequencing. Single cells were isolated with the BD Rhapsody Express Single-Cell Analysis System according to the manufacturer’s recommendations; after the final bead wash step, 40,000 cells were detected as singlets.

Libraries were prepared following the BD Rhapsody System mRNA Whole Transcriptome Analysis (WTA) and Sample Tag Library Preparation Protocol (BD Biosciences, 633801) and sequenced at the CCHMC Genomics Sequencing Facility on the NovaSeq 6000 using the PE (paired end) 100 sequencing format. After sequencing and preprocessing by the Rhapsody WTA Analysis Pipeline using exact cell count, 28,000 cells per cartridge were used for the downstream analysis.

### scRNA-Seq data analysis.

Raw scRNA-Seq data from 3 experimental conditions (naive, LNP, OVA-mRNA-LNP) were analyzed with v1.12.1 of the BD Rhapsody WTA Analysis Pipeline (BD Biosciences) on the Seven Bridges Platform (https://www.sevenbridges.com/platform). Sequencing reads in the FASTQ files were aligned to the GRCm38.p6 reference genome (GENCODE, mouse release M19). Cell calling was performed with the Refined Putative Cell Calling parameter disabled. Instead, the Exact Cell Count input was set to 28,000 cells. Generated feature-barcode matrices from 2 batches were merged, preserving the experimental condition and the batch information of each cell within its barcode. The merged feature-barcode matrix was then uploaded to SciDAP (https://scidap.com) for all subsequent data analysis steps.

Low-quality cells were removed with the scRNA-Seq Filtering Analysis pipeline (https://github.com/Barski-lab/workflows-datirium/blob/b210f5a5d3b303e3627582963a0b33b289f9a40e/workflows/sc-rna-filter.cwl) using the following quality control thresholds: a minimum of 500 transcripts per cell, 500–5,000 genes per cell, and maximum 5% of transcripts mapped to mitochondrial genes. Doublets were identified and removed with the scDblFinder R package (69) as part of this pipeline.

The remaining high-quality cells were processed by the scRNA-Seq Dimensionality Reduction Analysis pipeline (https://github.com/Barski-lab/workflows-datirium/blob/b210f5a5d3b303e3627582963a0b33b289f9a40e/workflows/sc-rna-reduce.cwl). Molecular count data were first corrected for technical variability and then integrated using the pairs of cells sharing a matched biological state as integration anchors. Other configuration parameters included (a) setting the normalization method to “sctglm,” which resulted in using the glmGamPoi R package within the SCTransform function (70); (b) limiting the number of highly variable genes for normalization and integration to 3,000; and (c) selecting the first 50 principal components (PCs) for principal component analysis and uniform manifold approximation and projection (UMAP) dimensionality reduction algorithms.

Dimensionally reduced single-cell data were clustered with the scRNA-Seq Cluster Analysis pipeline (https://github.com/Barski-lab/workflows-datirium/blob/b210f5a5d3b303e3627582963a0b33b289f9a40e/workflows/sc-rna-cluster.cwl) with 50 PCs and 1.0 clustering resolution. For each of the 27 obtained clusters, gene markers were identified using the FindAllMarkers function with default parameters (https://satijalab.org/seurat/reference/findallmarkers). The cluster with damaged cells (low transcripts-per-cell counts and nonspecific gene markers) was removed, and both scRNA-Seq Dimensionality Reduction Analysis and scRNA-Seq Cluster Analysis pipelines were rerun with 50 PCs and 1.0 clustering resolution. The resulting clusters produced cell types that were used in the following steps of the analyses.

Cells belonging to the T cell cluster were separated and reclustered by application of scRNA-Seq Dimensionality Reduction Analysis and scRNA-Seq Cluster Analysis pipelines. Dimensionality was reduced to 20 PCs, and the number of highly variable genes was limited to 1,000. The clustering resolution was set to 0.5.

Differentially expressed genes between experimental conditions were identified with the scRNA-Seq Differential Expression Analysis pipeline (https://github.com/Barski-lab/workflows-datirium/blob/b210f5a5d3b303e3627582963a0b33b289f9a40e/workflows/sc-rna-de-pseudobulk.cwl) run on the selected subsets of cells using the DESeq2 package (71). Gene expression data were aggregated to the pseudobulk level per sample.

Module scores for activated CD8^+^ T cells were calculated at the single-cell level, as previously described (48), using 197 genes identified in human CD8^+^ T cells following SARS-CoV-2 mRNA immunization (Supplemental Table 7) (49).

### Statistics.

The results are presented as mean ± SEM or displayed with box-and-whisker plots. Normality tests were applied to validate Gaussian distribution. Multiple-group comparisons were conducted using 1-way or 2-way analysis of variance (ANOVA) with Tukey correction applied. All statistical analyses, except scRNA-Seq analyses, were performed with Prism 10 software (GraphPad Software Inc.). *P* less than 0.05 was considered statistically significant (65–68).

### Study approval.

All experiments complied with protocols approved by the Cincinnati Children’s Hospital Medical Center (CCHMC) Animal Use and Care Committee.

### Data availability.

All data are available in the main text or the supplemental materials. Individual data point values can be found in the Supporting Data Values document. The single-cell RNA sequencing data were deposited at the NCBI’s Gene Expression Omnibus (GEO) repository under the accession number GSE259439.

## Author contributions

DW and MER supervised the study. YR, DW, and MER conceptualized the study. YR, MK, JMF, JMC, IPL, MGA, JRM, MR, AB, and DW developed methodology. YR, DW, and MER performed investigation. AMK, MR, JMC, GAO, JMF, LEM, and JH provided technical assistance. YR, MK, GAO, JH, IPL, AB, and MR performed analysis. DW and MER acquired funding. YR, DW, and MER wrote the original draft of the manuscript. YR, MK, MR, JRM, IPL, DW, and MER reviewed and edited the manuscript.

## Funding support

This work is the result of NIH funding, in whole or in part, and is subject to the NIH Public Access Policy. Through acceptance of this federal funding, the NIH has been given a right to make the work publicly available in PubMed Central.

- Food Allergy Fund.
- NIH/National Institute of Diabetes and Digestive and Kidney Diseases grant P30DK078392 (Digestive Health Center).

## Supplementary Material

Supplemental data

Supplemental table 2

Supplemental table 3

Supplemental table 4

Supplemental table 5

Supplemental table 6

Supporting data values

## Version 1 09/23/2025

In-Press Preview

## Version 2 11/03/2025

Electronic publication

## References

[1] Lambrecht BN, Hammad H (2017). The immunology of the allergy epidemic and the hygiene hypothesis. Nat Immunol.

[2] Sampath V (2021). Food allergy across the globe. J Allergy Clin Immunol.

[3] Warren CM (2024). Epidemiology and the growing epidemic of food allergy in children and adults across the globe. Curr Allergy Asthma Rep.

[4] Holgate ST, Polosa R (2008). Treatment strategies for allergy and asthma. Nat Rev Immunol.

[5] Pavord ID (2018). After asthma: redefining airways diseases. Lancet.

[6] Papi A (2018). Asthma. Lancet.

[7] Durham SR, Shamji MH (2023). Allergen immunotherapy: past, present and future. Nat Rev Immunol.

[8] Dorofeeva Y (2021). Past, present, and future of allergen immunotherapy vaccines. Allergy.

[9] Burks AW (2012). Oral immunotherapy for treatment of egg allergy in children. N Engl J Med.

[10] PALISADE Group of Clinical Investigators (2018). AR101 oral immunotherapy for peanut allergy. N Engl J Med.

[11] Santos AF (2025). EAACI guidelines on the management of IgE-mediated food allergy. Allergy.

[12] Orcel B (1994). Oral immunization with bacterial extracts for protection against acute bronchitis in elderly institutionalized patients with chronic bronchitis. Eur Respir J.

[13] Hattinger E (2015). Prophylactic mRNA vaccination against allergy confers long-term memory responses and persistent protection in mice. J Immunol Res.

[14] Weiss R (2017). Generation and evaluation of prophylactic mRNA vaccines against allergy. Methods Mol Biol.

[15] Scheiblhofer S (2018). DNA and mRNA vaccination against allergies. Pediatr Allergy Immunol.

[16] Niederberger V (2018). Safety and efficacy of immunotherapy with the recombinant B-cell epitope-based grass pollen vaccine BM32. J Allergy Clin Immunol.

[17] Cox L (2021). Grand challenges in allergen immunotherapy. Front Allergy.

[18] Akinfenwa O (2021). Novel vaccines for allergen-specific immunotherapy. Curr Opin Allergy Clin Immunol.

[19] Penagos M, Durham SR (2022). Allergen immunotherapy for long-term tolerance and prevention. J Allergy Clin Immunol.

[20] Pardi N (2017). Zika virus protection by a single low-dose nucleoside-modified mRNA vaccination. Nature.

[21] Chaudhary N (2021). mRNA vaccines for infectious diseases: principles, delivery and clinical translation. Nat Rev Drug Discov.

[22] Alameh MG (2022). Messenger RNA-based vaccines against infectious diseases. Curr Top Microbiol Immunol.

[23] Arevalo CP (2022). A multivalent nucleoside-modified mRNA vaccine against all known influenza virus subtypes. Science.

[24] Whitaker JA (2023). mRNA vaccines against respiratory viruses. Curr Opin Infect Dis.

[25] Lorentzen CL (2022). Clinical advances and ongoing trials on mRNA vaccines for cancer treatment. Lancet Oncol.

[26] Kon E (2023). Targeting cancer with mRNA-lipid nanoparticles: key considerations and future prospects. Nat Rev Clin Oncol.

[27] Krienke C (2021). A noninflammatory mRNA vaccine for treatment of experimental autoimmune encephalomyelitis. Science.

[28] Parhiz H (2024). mRNA-based therapeutics: looking beyond COVID-19 vaccines. Lancet.

[29] Kariko K (2005). Suppression of RNA recognition by Toll-like receptors: the impact of nucleoside modification and the evolutionary origin of RNA. Immunity.

[30] Kariko K (2008). Incorporation of pseudouridine into mRNA yields superior nonimmunogenic vector with increased translational capacity and biological stability. Mol Ther.

[31] Pardi N (2020). Recent advances in mRNA vaccine technology. Curr Opin Immunol.

[32] Alameh MG (2021). Lipid nanoparticles enhance the efficacy of mRNA and protein subunit vaccines by inducing robust T follicular helper cell and humoral responses. Immunity.

[33] Ndeupen S (2021). The mRNA-LNP platform’s lipid nanoparticle component used in preclinical vaccine studies is highly inflammatory. iScience.

[34] Verbeke R (2022). Innate immune mechanisms of mRNA vaccines. Immunity.

[35] Teijaro JR, Farber DL (2021). COVID-19 vaccines: modes of immune activation and future challenges. Nat Rev Immunol.

[36] Rohner E (2022). Unlocking the promise of mRNA therapeutics. Nat Biotechnol.

[37] Pardi N (2018). Nucleoside-modified mRNA vaccines induce potent T follicular helper and germinal center B cell responses. J Exp Med.

[38] Laczko D (2020). A single immunization with nucleoside-modified mRNA vaccines elicits strong cellular and humoral immune responses against SARS-CoV-2 in mice. Immunity.

[39] Xu X (2023). Use of a liver-targeting immune-tolerogenic mRNA lipid nanoparticle platform to treat peanut-induced anaphylaxis by single- and multiple-epitope nucleotide sequence delivery. ACS Nano.

[40] Bettini E, Locci M (2021). SARS-CoV-2 mRNA vaccines: immunological mechanism and beyond. Vaccines (Basel).

[41] Reber LL (2017). The pathophysiology of anaphylaxis. J Allergy Clin Immunol.

[42] Kanagaratham C (2020). IgE and IgG antibodies as regulators of mast cell and basophil functions in food allergy. Front Immunol.

[43] Han X (2018). Mapping the mouse cell atlas by Microwell-Seq. Cell.

[44] Grieshaber-Bouyer R (2021). The neutrotime transcriptional signature defines a single continuum of neutrophils across biological compartments. Nat Commun.

[45] Bain CC, MacDonald AS (2022). The impact of the lung environment on macrophage development, activation and function: diversity in the face of adversity. Mucosal Immunol.

[46] Fei L (2022). Systematic identification of cell-fate regulatory programs using a single-cell atlas of mouse development. Nat Genet.

[47] Wang R (2023). Construction of a cross-species cell landscape at single-cell level. Nucleic Acids Res.

[48] Tirosh I (2016). Dissecting the multicellular ecosystem of metastatic melanoma by single-cell RNA-seq. Science.

[49] Zhang B (2023). Multimodal single-cell datasets characterize antigen-specific CD8^+^ T cells across SARS-CoV-2 vaccination and infection. Nat Immunol.

[50] Enomoto N (2012). Allergen-specific CTL require perforin expression to suppress allergic airway inflammation. J Immunol.

[51] Rao RR (2010). The mTOR kinase determines effector versus memory CD8^+^ T cell fate by regulating the expression of transcription factors T-bet and Eomesodermin. Immunity.

[52] Powell JD, Delgoffe GM (2010). The mammalian target of rapamycin: linking T cell differentiation, function, and metabolism. Immunity.

[53] Li M (2006). Topical vitamin D3 and low-calcemic analogs induce thymic stromal lymphopoietin in mouse keratinocytes and trigger an atopic dermatitis. Proc Natl Acad Sci U S A.

[54] Shirinbak S (2010). Suppression of Th2-driven airway inflammation by allergen immunotherapy is independent of B cell and Ig responses in mice. J Immunol.

[55] Pham DL (2022). Characteristics of allergen profile, sensitization patterns and allergic rhinitis in SouthEast Asia. Curr Opin Allergy Clin Immunol.

[56] Zemelka-Wiacek M (2023). Hot topics in allergen immunotherapy, 2023: current status and future perspective. Allergy.

[57] Patil SU, Shreffler WG (2019). Novel vaccines: technology and development. J Allergy Clin Immunol.

[58] Tulaeva I (2020). Preventive allergen-specific vaccination against allergy: mission possible?. Front Immunol.

[59] Gamazo C (2017). Adjuvants for allergy immunotherapeutics. Hum Vaccin Immunother.

[60] Wang YH (2010). A novel subset of CD4(+) T(H)2 memory/effector cells that produce inflammatory IL-17 cytokine and promote the exacerbation of chronic allergic asthma. J Exp Med.

[61] Wang Y, Liu L (2024). Immunological factors, important players in the development of asthma. BMC Immunol.

[62] Griseri T (2015). Granulocyte macrophage colony-stimulating factor-activated eosinophils promote interleukin-23 driven chronic colitis. Immunity.

[63] Gomez Medellin JE (2025). Liver-targeted allergen immunotherapy rapidly and safely induces antigen-specific tolerance to treat allergic airway disease in mice. Sci Transl Med.

[64] Alameh MG (2024). A multivalent mRNA-LNP vaccine protects against *Clostridioides difficile* infection. Science.

[65] Baiersdorfer M (2019). A facile method for the removal of dsRNA contaminant from in vitro-transcribed mRNA. Mol Ther Nucleic Acids.

[66] Maier MA (2013). Biodegradable lipids enabling rapidly eliminated lipid nanoparticles for systemic delivery of RNAi therapeutics. Mol Ther.

[67] Zuo L (2010). IL-13 induces esophageal remodeling and gene expression by an eosinophil-independent, IL-13R alpha 2-inhibited pathway. J Immunol.

[68] Townsend JM (2000). IL-9-deficient mice establish fundamental roles for IL-9 in pulmonary mastocytosis and goblet cell hyperplasia but not T cell development. Immunity.

[69] Germain PL (2021). Doublet identification in single-cell sequencing data using *scDblFinder*. F1000Res.

[70] Choudhary S, Satija R (2022). Comparison and evaluation of statistical error models for scRNA-seq. Genome Biol.

[71] Love MI (2014). Moderated estimation of fold change and dispersion for RNA-seq data with DESeq2. Genome Biol.

