# Supplementary material for: Allergen-specific mRNA–lipid nanoparticle therapy for prevention and treatment of experimental allergy in mice
Source: J Clin Invest. 2025 Sep 23;135(21):e194080. doi: 10.1172/JCI194080 (PMC12578384; doi:10.1172/JCI194080)
Supplement: Supplemental data [file jci-135-194080-s258.pdf]

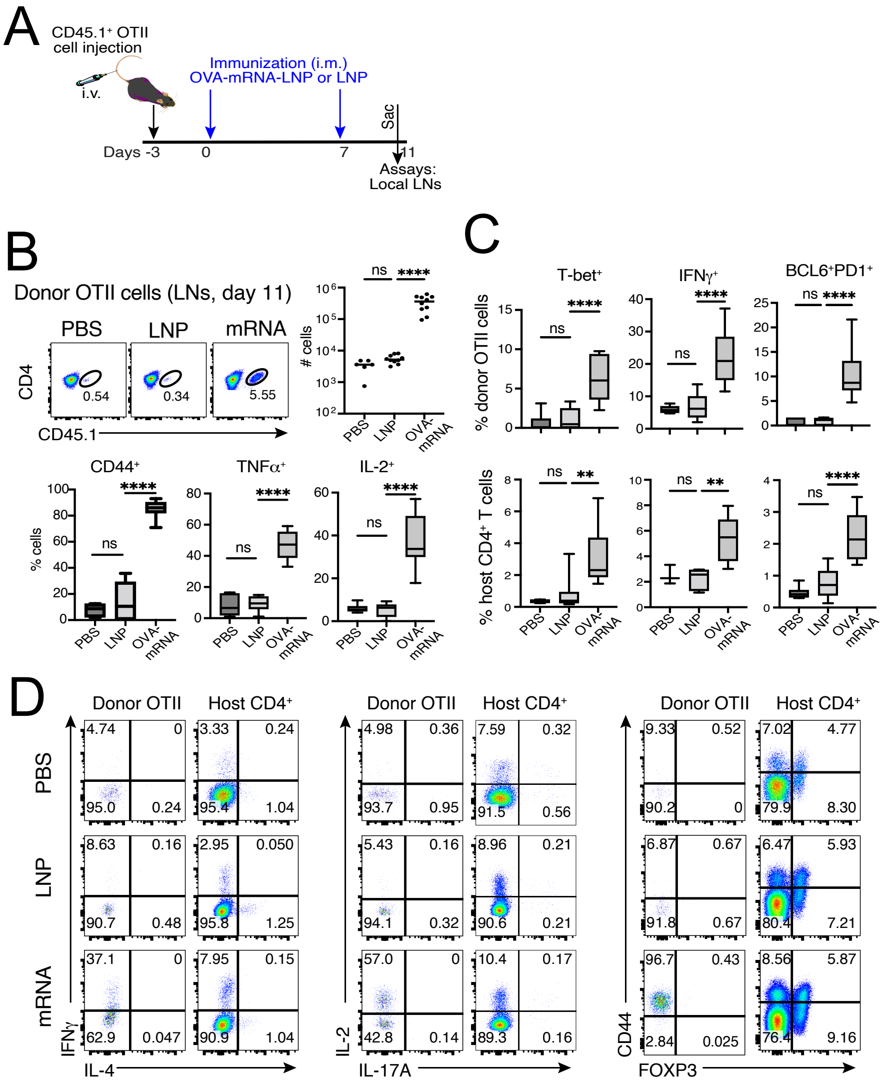

Supplemental Figure 1. The OVA-mRNA-LNP induces antigen-specific CD4$^+$ T cell expansion and differentiation toward Th1 and Tfh phenotypes.

(**A**) Schematic of the experimental design. Naive OTII cells expressing CD45.1 were injected intravenously (i.v.) into CD45.2 wild type mice 3 days prior to empty LNP or OVA-mRNA-LNP administration (day -3). The LNP or OVA-mRNA-LNP was delivered intramuscularly (i.m.) on days 0 and 7 (5 μg per mouse, blue arrows). Local lymph nodes (LNs) were collected on day 11. (**B**) The percent of donor CD45.1$^+$ OTII cells gated on CD4$^+$ T cell population and frequency of CD44$^+$, TNFα$^+$, and IL-2$^+$ cells gated on OTII cells were analyzed by flow cytometry. The number of donor cells within the local LNs of recipients was quantified. (**C**) Frequencies of donor or host CD4$^+$ T cells expressing specific markers are shown. The data are displayed as Box and Whiskers plots. (**D**) Representative flow cytometry plots of donor OTII cells (CD45.1$^+$) and host CD4$^+$

($CD45.2^+$) T cells producing indicated cytokines after short ex vivo stimulation and exhibiting CD44 and Foxp3. Numbers indicate cell frequencies. (B and C) Data from 3 independent experiments are presented (n = 6–10). $^{**}P \leq 0.01$, $^{****}P \leq 0.0001$ by one-way ANOVA with Tukey correction; ns, not significant. LNP, lipid nanoparticle; OVA, ovalbumin.

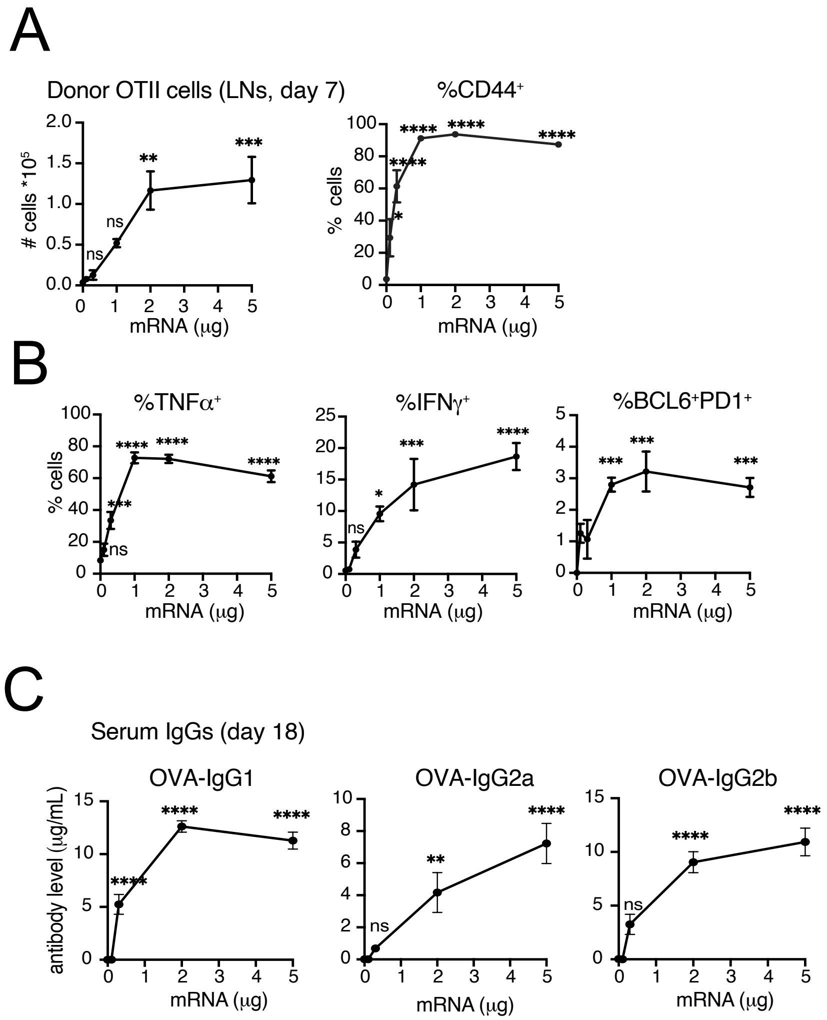

Supplemental Figure 2. A dose-response to OVA-mRNA-LNP treatment.

CD45.2 wild type mice were injected intravenously (i.v.) with naive OTII cells expressing CD45.1, followed by a single i.m. injection with OVA-mRNA-LNP (mRNA) in different concentrations. (**A** and **B**) A dose-response analysis of donor CD45.1$^+$ OTII cells in the lymph nodes (LNs) following a single OVA-mRNA-LNP injection (n = 3–4). Shown are results from 1 of 3 representative experiments on day 7 post vaccination. (**C**) OVA-specific IgG levels in the serum of the mice 18 days after first immunization with different concentrations of OVA-mRNA-LNP (n = 4–5). The experiment was repeated more than 5 times. (A–C) Data are mean ± SEM. *P ≤ 0.05, **P ≤ 0.01, ***P ≤ 0.001, ****P ≤ 0.0001 by one-way ANOVA; ns, not significant. LNP, lipid nanoparticle; OVA, ovalbumin.

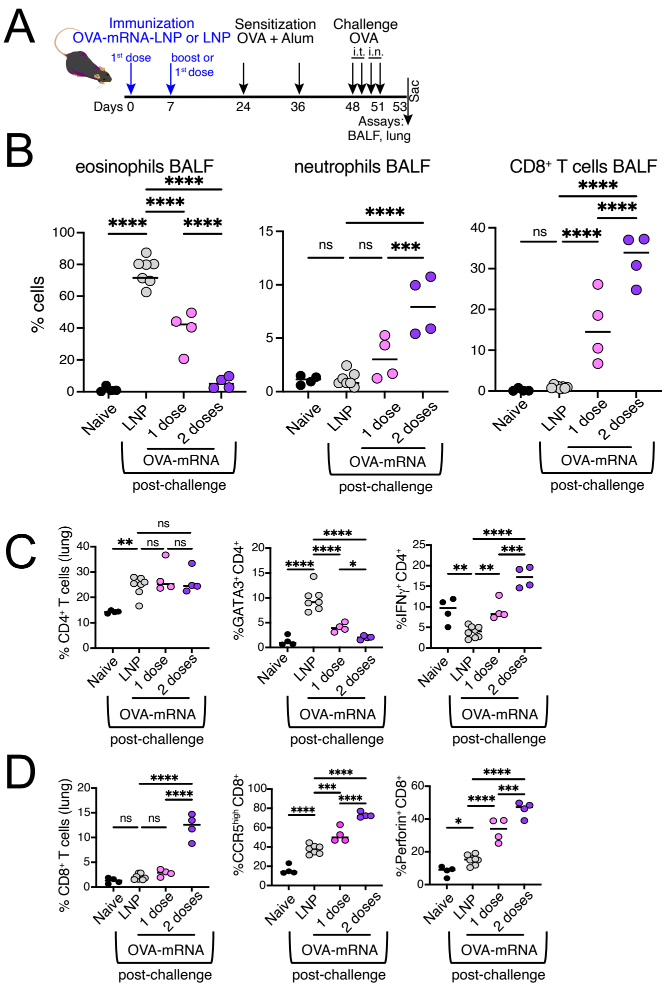

Supplemental Figure 3. OVA-mRNA-LNP booster increases anti-allergic responses.

(A) Experimental workflow. Mice received either 1 or 2 doses of the OVA-mRNA-LNP (mRNA) or empty LNP (LNP) administered 1 week apart (2 μg per injection, blue arrows) followed by a sensitization (days 24 and 36) and airway challenge protocol (intratracheal (i.t.) injection on days 48 and 49 and intranasal (i.n.) injection on days 50 and 51). Mice who received a single dose were vaccinated on day 7 for consistency. (**B**) Frequencies of eosinophils, neutrophils, and $CD8^+$ T cells in the bronchoalveolar lavage fluid (BALF) (n = 4–7). (**C**) Frequencies of indicated $CD4^+$ T cells or (**D**) $CD8^+$ T cells in the lungs (n = 4–7). (B–D) The data are presented as the mean, with each circle representing an individual sample. Shown is 1 of the 3 replicated experiments. $^*P \leq 0.05$, $^{**}P \leq 0.01$, $^{***}P \leq 0.001$, $^{****}P \leq 0.0001$ by one-way ANOVA with Tukey correction; ns, not significant. LNP, lipid nanoparticle; OVA, ovalbumin.

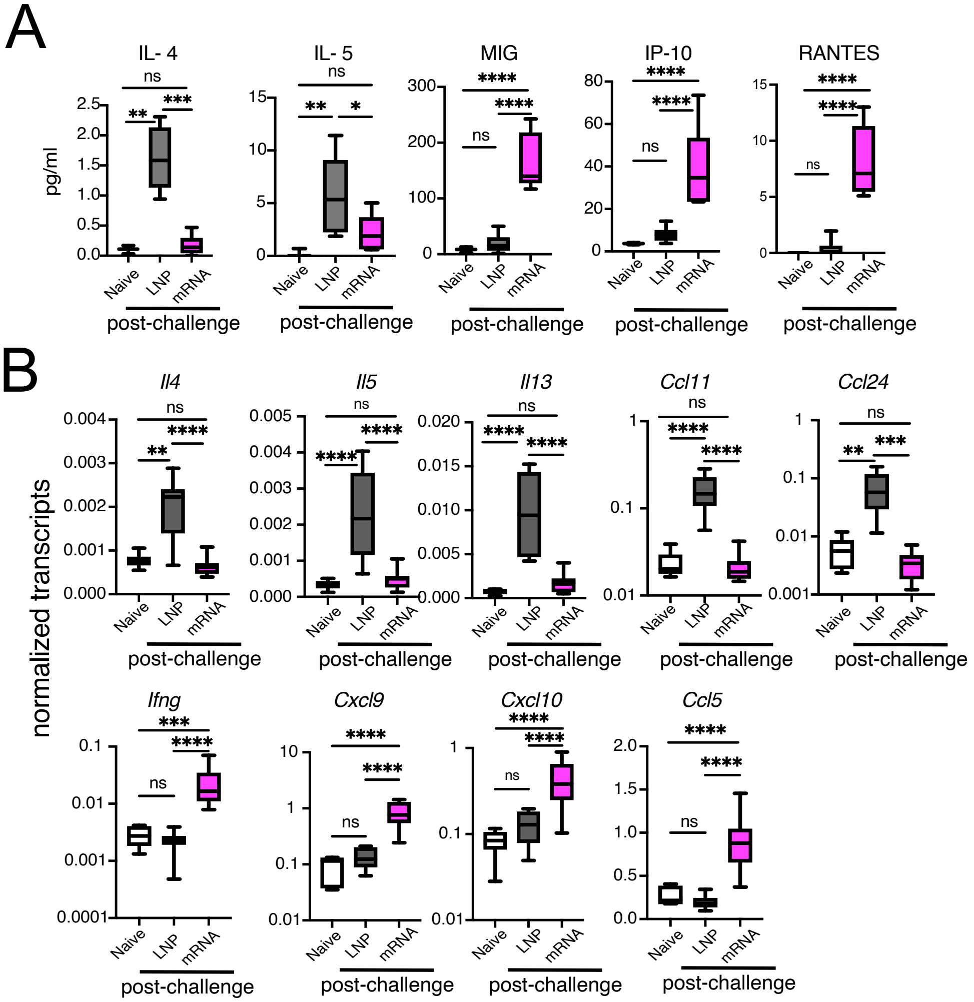

Supplemental Figure 4. Pulmonary cytokine expression upon OVA challenge.

Mice received 2 doses of empty LNP (LNP) or OVA-mRNA-LNP (mRNA) on days 0 and 7, followed by sensitization with OVA+Alum on days 24 and 36. Mice then were challenged with OVA for 4 consecutive days (days 48–51). Mice were sacrificed 2 days after the final challenge (day 53). Naïve mice served as unmanipulated controls. Bronchoalveolar lavage fluid (BALF) and lung tissue were collected 2 days after the final challenge (day 53). Naïve mice served as unmanipulated controls. (**A**) Protein level of indicated analytes in the BALF. (**B**) Quantitative PCR (qPCR) analysis of the specified transcripts expressed in the lung tissue and normalized to the housekeeping gene (*Eif3k*). Box and Whiskers plots (n = 6–15). *P ≤ 0.05, **P ≤ 0.01, ***P ≤ 0.001, ****P ≤ 0.0001 by one-way ANOVA with Tukey correction; ns, not significant. LNP, lipid nanoparticle; OVA, ovalbumin.

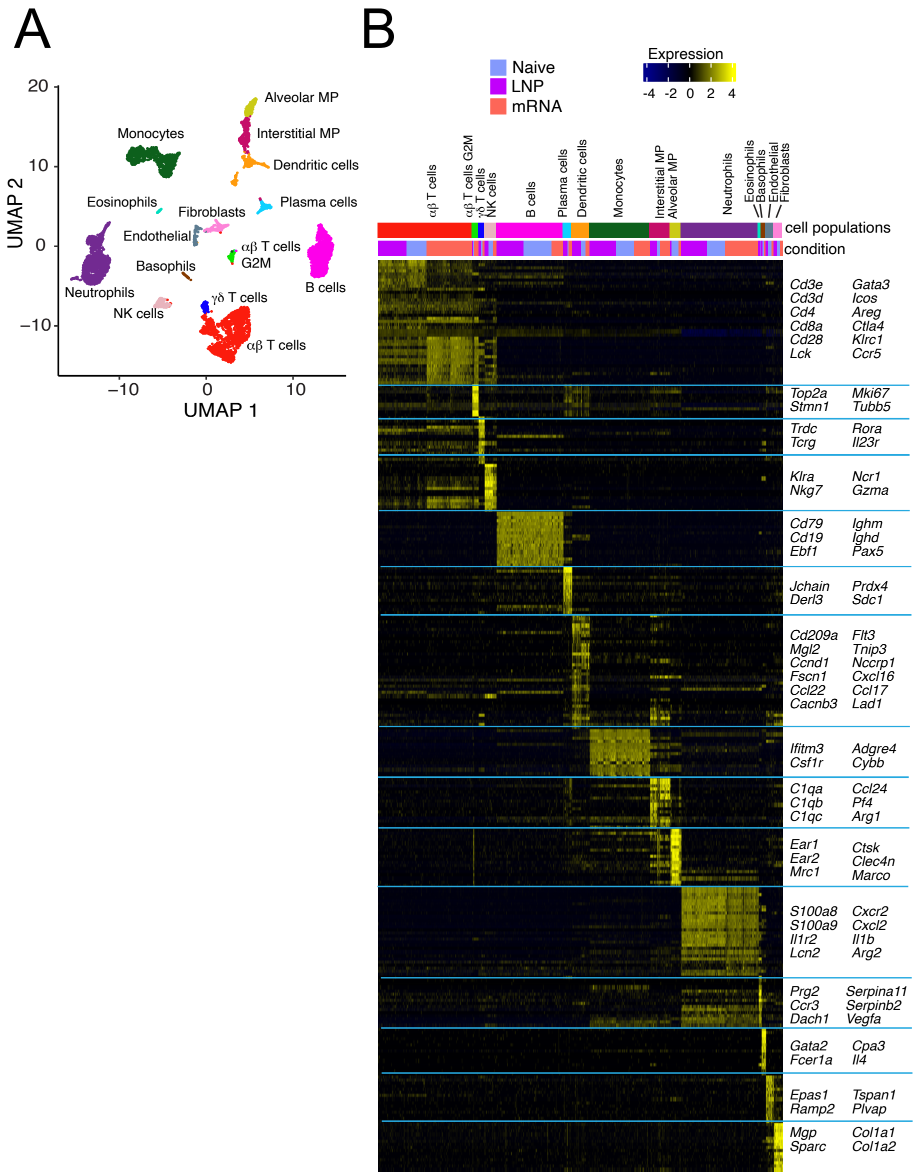

Supplemental Figure 5. Pulmonary scRNA-seq analysis upon OVA challenge.

Lung tissue from Naïve (unmanipulated), LNP, and OVA-mRNA-LNP mice was harvested 2 days after the final allergen challenge (day 53) and subjected to Rhapsody single-cell RNA sequencing (scRNA-seq). (**A**) Uniform manifold approximation and projection (UMAP) plot illustrating 15 cell clusters identified by unsupervised clustering, encompassing 4 different conditions (n = 2). (**B**) Heatmap of genes representing each cell population (shown are the top of highly expressed genes with adjusted $P < 0.05$). MP, macrophages; NK, natural killer; Treg, regulatory T cell; G2M, growth 2- mitosis.

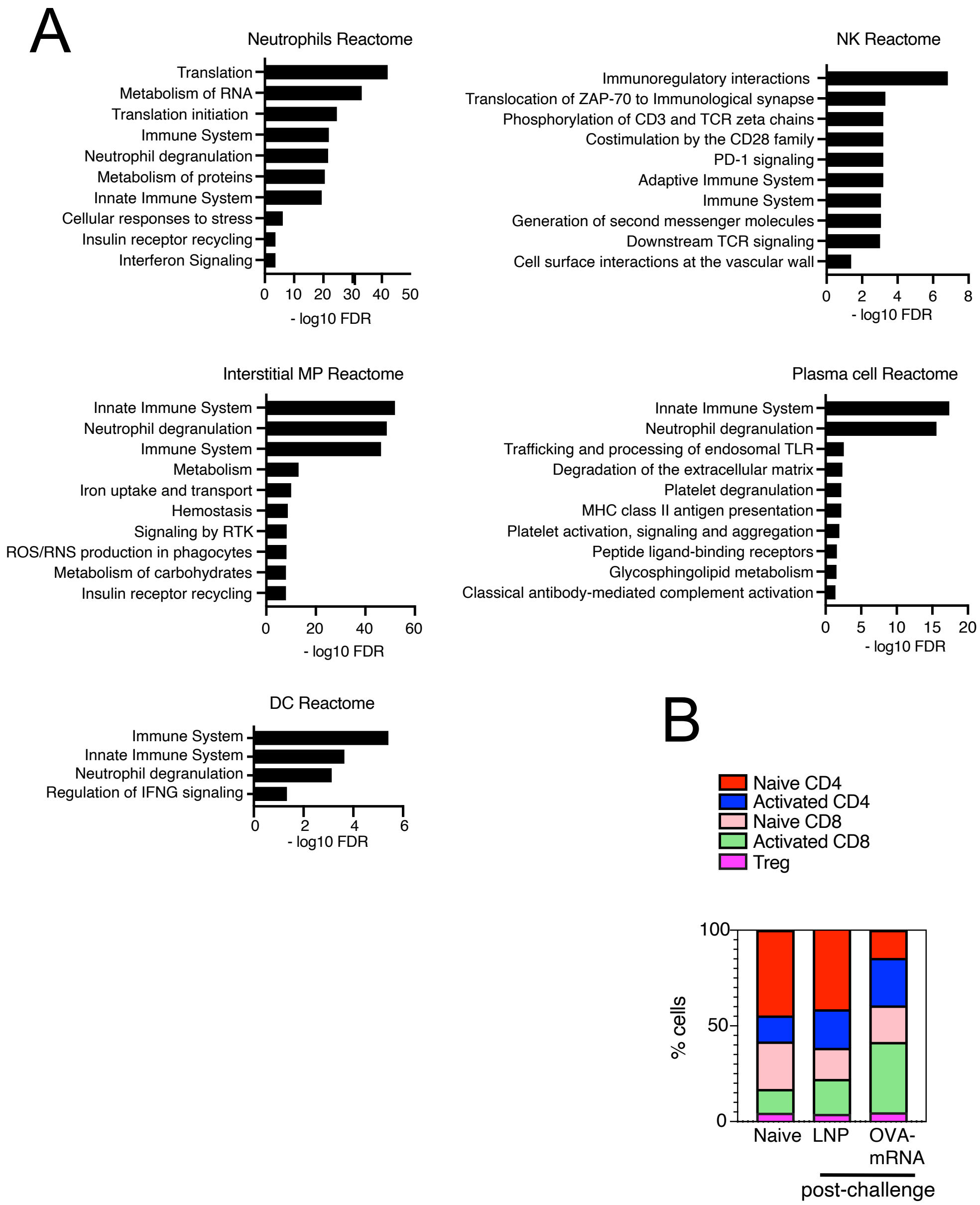

Supplemental Figure 6. Pulmonary transcriptional pathway analysis post-OVA Challenge. Lung tissue from naïve (unmanipulated), LNP, and OVA-mRNA-LNP-treated mice was collected 2 days after the final allergen challenge and subjected to Rhapsody single-cell RNA sequencing (scRNA-seq). (**A**) Differential gene expression (DEG) analysis was performed across the indicated cell populations comparing the LNP and OVA-mRNA-LNP groups, using the following criteria: at least 35% of cells expressing the gene in one of the conditions, P adjusted < 0.05, and a fold change >1.5. Identified DEGs (Supplemental Tables 2-6) were further analyzed using Reactome pathway analysis. (**B**) Distribution of T cell subtypes by treatment condition was assessed based on clustering analysis of scRNA-seq data.

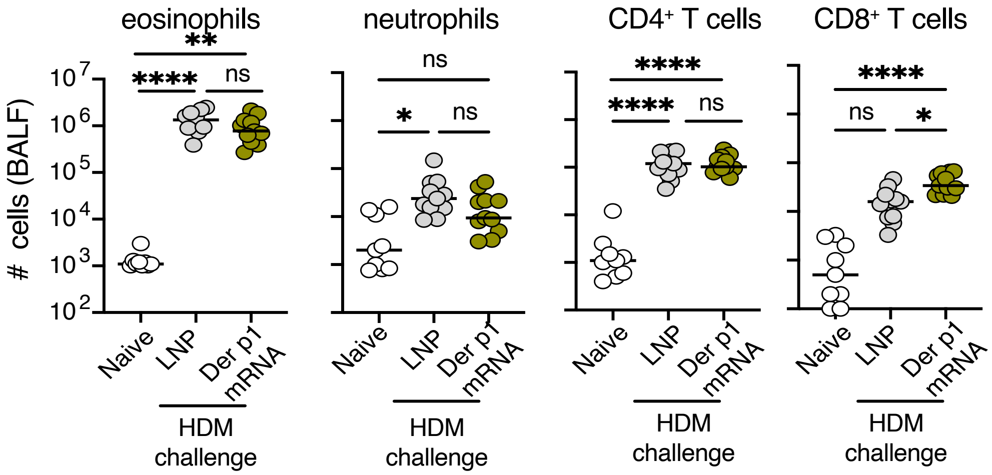

Supplemental Figure 7. BALF cell count upon HDM challenge.

Mice were injected with LNP or Der p1-mRNA-LNP on days 0 and 7, and both groups were sensitized with HDM and challenged with HDM extract intranasally. Shown is the quantification of cells in the bronchoalveolar lavage fluid (BALF) on day 2 after the last challenge combined from 3 independent experiments (n = 9–12). Each circle represents an individual sample; data are shown as mean ± SEM. *P ≤ 0.05, **P ≤ 0.01, ****P ≤ 0.0001 by one-way ANOVA with Tukey correction; ns, not significant. HDM, house dust mite; LNP, lipid nanoparticle.

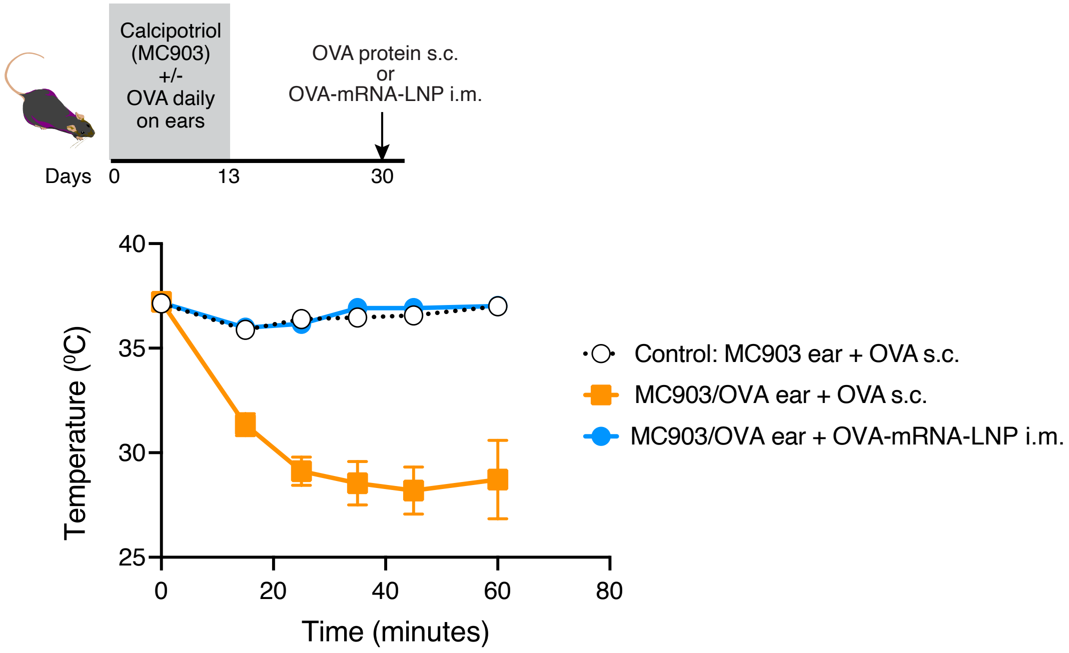

Supplemental Figure 8. Temperature changes following allergen challenge in mice treated with allergen-specific mRNA-LNP or subcutaneous immunotherapy.

Mice were cutaneously sensitized with OVA in the presence of calcipotriol (MC903) daily in the ears for 2 weeks. Control mice were treated with calcipotriol without OVA. Two and a half weeks later, mice were treated subcutaneously (s.c.) with 1 mg endotoxin-free OVA or intramuscularly (i.m.) with 10 µg OVA-mRNA-LNP. Temperature changes were measured at the indicated time points post treatment (n = 4–5). Data are presented as mean ± SD. Shown is 1 of the 2 replicated experiments. LNP, lipid nanoparticle; OVA, ovalbumin.

pg/ml	Naive1	Naive2	Naive3	LNP1	LNP2	LNP3	LNP4	LNP5	LNP6	mRNA1	mRNA2	mRNA3	mRNA4	mRNA5	mRNA6	RNA7
IL-4	0.11	0.03	0.17	1.84	2.06	1.21	1.33	0.94	2.31	0.05	0.11	0.27	0.47	0.06	0.17	0.29
IL-5	0.7	0	0	8.23	6.21	4.47	2.43	1.87	11.42	3.31	3.7	1.52	2.23	5.02	0.84	0.62
KC	24.01	13.33	13.47	44.41	57.63	52.67	17.05	22.32	51.12	45.81	17.18	23.05	25.19	33.23	26.13	38.46
G-CSF	11.72	4.01	2.12	15.56	23.84	16.2	6.72	7.34	19.87	55.53	135.8	9.75	154.13	15.07	29.44	7.95
IL-6	2.27	0.89	0.48	5.77	6.19	5.99	1.1	2.35	8.95	16.84	5.68	6.7	122.62	40.21	12.15	7.23
MCP-1	4.45	0.5	1.32	1.95	4.45	3.83	1.32	0.69	1.32	100.44	9.49	1.95	263.61	213.19	39.79	12.06
IP-10	3.7	3.17	4.14	5.93	8.34	14.21	3.77	7.42	7.8	37.88	34.62	23.38	73.55	53.27	23.63	34.72
MIG	2.43	8.7	12.89	9.48	21.48	50.16	1.69	9.37	22.38	128.6	128.28	116.9	242.67	217.49	139.75	204.92
MIP-1a	3.85	0	0	3.85	12.27	18.66	0	12.27	3.85	122.72	81.91	25.71	149.93	100.73	75.89	30.05
MIP-1b	0	0	0	0	0	0	0	0	0	59.56	41.78	0	122.21	31.61	37.25	68.52
TNFa	0	0	0	0	0	0	0	0	0	2.89	1.01	0	7.12	1.69	0.3	3.59
LIF	0.58	0.29	0.2	2.81	2.77	2.51	1.4	1.65	1.91	9.88	2.81	0.83	24.42	38.08	17.29	1.78
IFNg	0.24	0.32	0	0	0.39	0.39	0	0.39	0	1.16	0.09	0.09	4.44	1.69	0	7.02
RANTES	0	0	0	0	0	1.96	0.16	0	0	7.1	5.51	6.22	11.26	11.1	13.01	28.25

Supplemental Table 1. Table summarizing the multiplex cytokine analysis of the bronchoalveolar lavage fluid (BALF). LNP, lipid nanoparticle; mRNA, OVA-mRNA-LNP.

Gene name				
abracl	chmp2a	gzmb	nt5c3a	taldo1
ac010618.1	cldnd1	gzmh	nudt5	tap1
acaa2	clic1	h3f3a	oas2	tceal8
actb	clic3	hadhb	ostf1	timd4
actg1	clta	havcr2	park7	tkt
actr3	cnn2	hla-dma	pclaf	tmem256
ada	commd4	hla-dra	pfdn4	tmsb10
anxa5	copb2	hmga1	pfn1	tmsb4x
ap1s1	cops9	ica1	polr2g	tpm4
ap2s1	copz1	idh2	pomp	trappc1
apobec3c	coro1a	ifi16	ppp1ca	troap
apobec3g	cotl1	ifi27l2	ppp1r18	twf2
apobec3h	cox5a	igbp1	ppp4c	txndc17
arhgap30	cox6b1	irf4	prf1	txndc9
arhgdib	cox6c	isg15	psma2	ube2l6
arpc1b	csk	itgb1	psma4	ucp2
arpc2	cxcr3	jpt1	psma5	wdr1
arpc3	dbi	lair2	psmb2	yars
arpc4	dctn2	lamtor2	psmb3	ywhae
arpc5	dctn3	lap3	psmb4	znhit1
atp5f1a	decr1	lcp1	psmb8	zyx
atp5f1b	dnajc15	ldhb	psmd8	
atp5f1c	dynll1	lgals1	psme1	
atp5mc2	eif2s2	limd2	ptrhd1	
atp5mc3	eif4e2	mpg	pttg1	
atp5mf	ezh2	mrpl10	pycard	
atp5mg	fabp5	mrpl28	qars	
atp6v1d	fermt3	mrpl42	rabl3	
bcl2l11	fgfbp2	mrpl51	rac2	
bloc1s1	fibp	mt-co1	rack1	
calm3	fkbp1a	mt1e	rgs10	
capzb	fkbp3	mt1f	rpa3	
card16	gapdh	mxd4	rps6ka1	
carhsp1	gbp1	myl6	rtraf	
ccdc28b	gbp2	myl6b	s1pr4	
ccl5	ggct	nckap1l	sec11a	
cd27	gimap1	ncoa4	sem1	
cd38	gimap4	ndufa12	serpinb1	
cd52	glrx	ndufb3	sh3bgrl3	
cebpd	gmfg	ndufb5	sit1	
cfl1	gsto1	ndufc2	slc25a5	
chchd1	gtf2h5	ndufs2	srsf9	
chchd5	gtf3c6	nop10	sub1	
chi3l2	gzma	nsmce1	suclg1	

Supplemental Table 7. The human gene set included 197 genes identified in CD8 T cells following SARS-CoV-2 mRNA-LNP vaccination *(49).*

REAGENT	SOURCE	IDENTIFIER
**Reagents**		
Liberase TL	Sigma/Roche	Catalog #: 5401020001
Deoxyribonuclease I (DNaseI)	Sigma-Aldrich	Catalog #: DN25
Formalin solution, neutral buffered, 10%	Sigma-Aldrich	Catalog#: HT501128
Phorbol 12,13-dibutyrate	Sigma-Aldrich	Catalog #: P1269
Ionomycin	Sigma-Aldrich	Catalog #: I0634
Albumin from chicken egg white	Sigma-Aldrich	Catalog #: A5503
TriPure™ Isolation Reagent	Sigma-Aldrich Roche	Catalog #: 11667165001
Brefeldin A Solution (1000X)	ThermoFisher	Catalog #: 00-4506-51
Imject ALUM	ThermoFisher	Catalog #: 77161
SuperBlock™ Blocking Buffer	ThermoFisher	Catalog #: 37515
eBioscience™ Foxp3 / Transcription Factor Fixation/Permea	ThermoFisher	Catalog #: 00-5521-00
PowerUp™ SYBR™ Green Master Mix for qPCR	ThermoFisher	Catalog #: A25742
ACK lysing buffer	Quality Biological	Catalog #: 118-156-101
BD Microtainer	BD Biosciencies	Catalog #: 365967
Cell strainer 70 mm	BD Biosciencies	Catalog #: 352350
BD OptEIA™ TMB Substrate Reagent Set	BD Biosciencies	Catalog #: 555214
Streptavidin-HRP	RnD Systems	Catalog #: DY998
Everolimus	Tocris	Catalog #: 6188
Calcipotriol MC903	Tocris	Catalog #: 112965-21-6
Natural Der p 1 (np-Der p1)	inbio	Catalog #: NA-DP1-1
Quick-RNA Miniprep Kit	Zymo Research	Catalog #: R1054
ProtoScript cDNA synthesis kit	New England BioLabs	Catalog #: e6300
EasySep™ Mouse Naïve CD4+ T Cell Isolation Kit	STEMCELL Technologies	Catalog #: 19765
Aluminum hydroxide gel	InVivoGen	Catalog #: vac-alu-50
Ovalbumin EndoFit	InVivoGen	Catalog #: vac-pova
House Dust mite extract	GREER laboratories Inc.	Catalog #: XPB82D3A2.5
**Antibodies**		
Mouse CCL24/Eotaxin-2/MPIF-2 DuoSet	R&D Systems	Catalog #: DY528
HRP Rat Anti-Mouse IgG1	BD Bioscience	Catalog #: 559626
HRP Rat Anti-Mouse IgG2a	BD Bioscience	Catalog #: 553391
Biotin Rat Anti-Mouse IgG2b	BD Bioscience	Catalog #: 553393
Purified anti-chicken Ovalbumin Antibody (IgG1)	Biolegend	Catalog #: 520501
Purified anti-chicken Ovalbumin Antibody (IgG2a)	Biolegend	Catalog #: 520401
LEGEND MAX™ Mouse OVA Specific IgE ELISA Kit	BioLegend	Catalog #: 439807
Mouse Anti-Ovalbumin IgG2b Monoclonal antibody	Chondrex	Catalog #: 7096
Mouse Anti-HDM Der p1 IgG1 Antibody Assay Kit	Chondrex	Catalog #: 3048
Purified Rat Anti-Mouse CD16/CD32 (Mouse BD Fc Block™	BD Bioscience	Catalog #: 553142
Alexa Fluor® 647 Mouse anti-Bcl-6	BD Bioscience	Catalog #: 561525
Fixable viability dye eFluor 780	ThermoFisher	Catalog #: 65-0865-18
Brilliant Violet 510™ anti-mouse CD4	BioLegend	Catalog #: 100559
Brilliant Violet 785™ anti-mouse CD8a	BioLegend	Catalog #: 100749
Brilliant Violet 605™ anti-mouse CD45.1	BioLegend	Catalog #: 110738
APC anti-mouse CD45.2	BioLegend	Catalog #: 109814
FITC anti-mouse/human CD44	BioLegend	Catalog #: 103006
Brilliant Violet 421™ anti-mouse CD25	BioLegend	Catalog #: 102043
Brilliant Violet 785™ anti-mouse CD279 (PD-1)	BioLegend	Catalog #: 135225
PE/Cyanine7 anti-T-bet	BioLegend	Catalog #: 644823
PE/Cyanine7 anti-mouse CD195 (CCR5)	BioLegend	Catalog #: 107017
PE/Cyanine7 anti-mouse/human KLRG1	BioLegend	Catalog #: 138416
Brilliant Violet 421™ anti-mouse CD38	BioLegend	Catalog #: 102732
PE anti-mouse CD170 (Siglec-F)	BioLegend	Catalog #: 155506
Alexa Fluor® 488 anti-mouse CD11c	BioLegend	Catalog #: 117311
Brilliant Violet 421™ anti-mouse/human CD11b	BioLegend	Catalog #: 101236
Alexa Fluor® 700 anti-mouse/human CD11b	BioLegend	Catalog #: 101222
Brilliant Violet 605™ anti-mouse CD64 (FcγRI)	BioLegend	Catalog #: 139323
PE/Dazzle™ 594 anti-mouse CD64	BioLegend	Catalog #: 139320
PE/Cyanine7 anti-mouse Ly-6G/Ly-6C (Gr-1)	BioLegend	Catalog #: 108416
PE/Cyanine7 anti-mouse Ly-6G	BioLegend	Catalog #: 127618
APC/Cyanine7 anti-mouse CD3ε	BioLegend	Catalog #: 100330
APC anti-mouse IL-4 Antibody	BioLegend	Catalog #: 504106
Brilliant Violet 711™ anti-mouse IFN-γ	BioLegend	Catalog #: 505836
PerCP/Cyanine5.5 anti-mouse IL-2	BioLegend	Catalog #: 503821
Brilliant Violet 510™ anti-mouse TNF-α	BioLegend	Catalog #: 506339
PE/Dazzle™ 594 anti-mouse Perforin	BioLegend	Catalog #: 154315
PE/Dazzle™ 594 anti-mouse IL-17A	BioLegend	Catalog #: 506937
APC anti-mouse IL-17A	BioLegend	Catalog #: 506915
Brilliant Violet 605™ anti-mouse IL-33Rα (IL1RL1, ST2)	BioLegend	Catalog #: 145323
PE anti-mouse CD186 (CXCR6)	BioLegend	Catalog #: 151103
FOXP3 Monoclonal Antibody (FJK-16s), Alexa Fluor 700	ThermoFisher	Catalog #: 56-5773-82
IL-5 Monoclonal Antibody (TRFK5), PE	ThermoFisher	Catalog #: 12-7052-82
IL-13 Monoclonal Antibody (eBio13A), eFluor 450	ThermoFisher	Catalog #: 48-7133-82
Gata-3 Monoclonal Antibody (TWAJ), PE,	ThermoFisher	Catalog #: 12-9966-42
Gata-3 Monoclonal Antibody (TWAJ), PerCP-eFluor™ 710	ThermoFisher	Catalog #: 46-9966-42
CD25 Monoclonal Antibody (PC61.5), PE-eFluor™ 610	ThermoFisher	Catalog #: 61-0251-82
CD45 Monoclonal Antibody (30-F11), PerCP-Cyanine5.5	ThermoFisher	Catalog #: 45-0451-82

Supplemental Table 8.
Reagents and Antibodies

gene	Forward primer	Reverse primer
Il4	ATGGAGCTGCAGAGACTCTTTC	TGGACTCATTCATGGTGCAG
Il5	ATGAGGCTTCCTGTCCCTAC	CCACGGACAGTTTGATTCTTC
Il13	TGTGTCTCTCCCTCTGACCC	GCCAGGTCCACACTCCATAC
Ifng	ACAGCAAGGCGAAAAAGGAT	GAGCTCATTGAATGCTTGGC
Cxcl9	GCCCCAATTGCAACAAAACT	GCTTCTTCACATTTGCCGAG
Cxcl10	AGTGCTGCCGTCATTTTCTG	ATTCTCACTGGCCCGTCATC
Ccl5	CATCATCCTCACTGCAGCC	GAGGGAGAGGTAGGCAAAGC
Ccl11	CACGGTCACTTCCTTCACCT	TGGGGATCTTCTTACTGGTCA
Ccl24	AGCATCTGTCCCAAGGCAG	CTCTGAACCCACAGCAGCTT
Eif3k	CACAAGCCAAAGAGAATGCC	TGAGGGCTTTCAGCAGAATC

Supplemental Table 9.
Real-Time PCR primers.
